# A First Tentative for Simultaneous Detection of Fungicides in Model and Real Wines by Microwave Sensor Coupled to Molecularly Imprinted Sol-Gel Polymers

**DOI:** 10.3390/s20216224

**Published:** 2020-10-31

**Authors:** Jérôme Rossignol, Laurence Dujourdy, Didier Stuerga, Philippe Cayot, Régis D. Gougeon, Elias Bou-Maroun

**Affiliations:** 1 Laboratoire Interdisciplinaire Carnot de Bourgogne, CNRS UMR 6303, Departement Interface, GERM, University Bourgogne Franche-Comté, 21078 Dijon, France; jerome.rossignol@u-bourgogne.fr (J.R.); didier.stuerga@u-bourgogne.fr (D.S.); 2 Service d’Appui à la Recherche, AgroSup Dijon, F-21000 Dijon, France; laurence.dujourdy@agrosupdijon.fr; 3 AgroSup Dijon, University Bourgogne Franche-Comté, PAM UMR A 02.102, Procédés Alimentaires et Microbiologiques, F-21000 Dijon, France; philippe.cayot@agrosupdijon.fr (P.C.); regis.gougeon@u-bourgogne.fr (R.D.G.); 4 Institut Universitaire de la Vigne et du Vin Jules Guyot, AgroSup Dijon, University Bourgogne Franche-Comté, PAM UMR A 02.102, Procédés Alimentaires et Microbiologiques, F-21000 Dijon, France

**Keywords:** molecularly imprinted polymers, microwave sensor, chemometric methods, pesticides, wine, rapid detection

## Abstract

A molecularly imprinted silica (MIS) coupled to a microwave sensor was used to detect three fungicides (iprodione, procymidone and pyrimethanil) present in most French wines. Chemometric methods were applied to interpret the microwave spectra and to correlate microwave signals and fungicide concentrations in a model wine medium, and in white and red Burgundy wines. The developed microwave sensor coupled to an MIS and to its control, a nonimprinted silica (NIS), was successfully applied to detect the three fungicides present in trace levels (ng L^−1^) in a model wine. The MIS sensor discriminated the fungicide concentrations better than the NIS sensor. Partial Least Squares models were suitable for determining iprodione in white and red wines. A preliminary method validation was applied to iprodione in the white and red wines. It showed a limit of detection (LOD) lower than 30 ng L^−1^ and a recovery percentage between 90 and 110% when the iprodione concentration was higher than the LOD. The determined concentrations were below the authorized level by far.

## 1. Introduction

Grapevines are vulnerable to a wide range of fungal pathogens, including Botrytis bunch rot (Botrytis cinerea), powdery mildew (Uncinula necator), downy mildew (Plasmopara viticola), black rot (Gugnardia bidwelli) and several others [1]. To fight them, fungicides are widely used in agriculture. Vineyards represent 3.7% of the French agricultural surface but are responsible for about 20% of pesticide consumption, 80% of which are fungicides [2]. In France, grapes are one of the most treated fruits and therefore the most contaminated ones [3]. At the European level, a 2008 wide survey (PAN-EUROPE) of 34 conventional wines from eight countries showed that 100% of conventional analyzed wines contained between four and 10 different fungicide residues [4].

To protect the consumer, the European Union has set Maximum Residue Limits (MRLs) for pesticides in grapes at 0.02 mg/kg [5]. The grape MRL is used as a reference for wine since there is no MRL for the latter.

The control of pesticide residues in wine, and more generally in beverages (including water), requires sensitive, selective and inexpensive analytical methods. Currently, the detection methods used are mainly based on mass spectrometry detection and are relatively expensive [6,7,8]. In addition, these methods require a sample preparation step, which makes them time consuming. In parallel, ELISA methods using an immunosorbent are used [9,10]. Other than their high cost, ELISA methods cannot be used in extreme pH or temperature environments.

A cheap, fast and real-time analysis method that will improve food safety and quality is of great necessity. It will also have great economic benefits in comparison with the currently used methods, resulting in time saving and small equipment investment. In comparison with the chromatographic methods, the in-situ method will allow for solvents to be saved and wastes to be reduced. For this reason, in this study we used a microwave sensor coupled to molecularly imprinted polymers. The goal was to monitor, at room temperature and in real-time, some fungicides in model, white and red Burgundy wines.

Molecularly imprinted polymers (MIPs) are one of the most specific and selective materials. For the analysis of small metabolites, MIPs mimic the high specificity of an antibody toward its antigen, of an enzyme toward its substrate or of a receptor toward its hormone. MIPs are known to provide a high selectivity of interaction, similar to biological materials [11]. They were used in this study to interact with high specificity with the target fungicides. In addition, they showed an increased stability under extreme conditions of pH and temperature [12]. Finally, the big advantage of MIPs is their low cost of production [13].

Several transducing systems were already used in combination with molecularly imprinted polymers, such as: electro-chemiluminescence-[14], fluorescence-[15], surface plasmon resonance-[16], surface acoustic wave (SAW)-[17], electrochemical-[18], piezoelectric-[19], capacitance-[20] and thermometric-based [21] sensors. For small molecules such as antibiotics, both SAW and electrochemical sensors based on MIPs were successfully developed [22,23]. The use of a microwave signal in a broad range of frequencies (10 MHz and 20 GHz) provides fruitful information and data on the interaction between the target and the sensitive material in comparison with other transduction methods. This allows for a better recognition and quantification of the target.

Microwave spectra are complex due to dielectric relaxation in liquids and solids. Chemometric tools such as multivariate analysis can therefore be required for an improved interpretation of the spectra. In general, a microwave spectrum is not perfect, and as such it must be preprocessed prior to modelling. The main objective of data processing is to transform the spectrum into the best fit condition and to ensure that an optimal performance can be achieved in later stages [24,25,26].

In a previous study [27], the feasibility of a microwave sensor based on a molecular sol-gel polymer was demonstrated for the detection of iprodione fungicide in a hydroalcoholic medium. In this study, for an optimal interpretation of the results, multivariate data analyses were used to reduce the dimensionality of the experimental datasets. A microwave sensor coupled to molecularly imprinted silica (MIS) was used for the simultaneous detection of iprodione, procymidone and pyrimethanil fungicides in a model wine. Moreover, the detection of iprodione was demonstrated in white and red wines.

## 2. Materials and Methods

### 2.1. Chemicals and Samples

Iprodione (97%, CAS number 36734-19-7), (3-Aminopropyl)trimethoxysilane (APTMS 97%, CAS number 13822-56-5), tetraethoxysilane (TEOS ≥ 99%, CAS number 78-10-4), ammonium hydroxide (NH_4_OH 28–30%, CAS Number 1336-21-6), poly vinyl chloride (PVC, CAS Number 9002-86-2), tetrahydrofuran (THF 99.9%, CAS Number 109-99-9), absolute ethanol (≥99.8%, CAS Number 64-17-5) and acetonitrile for High Performance Liquid Chromatography (HPLC) (≥99.9%, CAS Number 75-05-8) were purchased from Sigma Aldrich, France. Procymidone (≥98%, CAS number 32809-16-8) and pyrimethanil (≥98%, CAS number 53112-28-0) were bought from Chemos GmbH, Regenstauf, Germany. S1813 photosensitive resin and MF319 developer were purchased from Chimie Tech Service. The water used in all experiments was deionized and obtained from an Elga Ionic system PURELAB Option. The model wine consisted of water/ethanol (90/10, *v*/*v*) solutions. A Chardonnay white wine from Burgundy (Mâcon–Village 2011) and red wine from Burgundy (Domaine Sorin de France 2014) were bought from a grocery store.

### 2.2. Preparation of the Molecularly Imprinted Sol-Gel Polymer

The molecularly imprinted polymer was synthesized using sol-gel polymerization respecting a molar ratio of (iprodione, APTMS, TEOS, NH_4_OH, ethanol, water) of (1, 4, 50, 100, 1.15, 0.44). This ratio was chosen based on the bibliography [28,29] and on some preliminary tests performed in the laboratory. After the solubilization of iprodione in an ethanol/water mixture at 40 °C under magnetic stirring, APTMS and then TEOS were respectively added. After addition of NH_4_OH, the solution became cloudy. The mixture was left under stirring for 20 h at 40 °C. The polymer was recovered as a powder after centrifugation. In order to eliminate iprodione, the polymer was washed several times with ethanol until no trace of iprodione was detected by reverse phase high performance liquid chromatography in the washing solution. After washing, the polymer was dried at 60 °C overnight and stored in a desiccator at ambient temperature until use.

A nonimprinted silica (NIS) serving as the control polymer was prepared under the same conditions as the molecularly imprinted silica (MIS) but without adding the template (iprodione) in the ethanol/water mixture.

### 2.3. Microwave Sensor and Measurement Conditions

**Figure 1:**
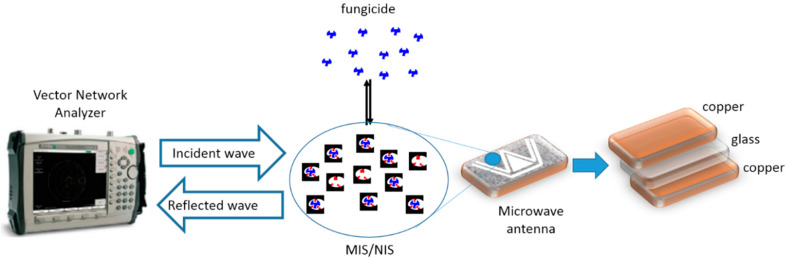
Microwave sensor showing an antenna recovered by a layer of molecularly imprinted silica (MIS) or nonimprinted silica (NIS). The VNA (Vector Network Analyzer) generates a wave between 10 MHz and 20 GHz and records the reflection coefficient (Γ(f)) at each frequency. The W geometry of the antenna was designed to enhance the microwave signal.

The microwave sensor coupled to MIS and NIS was described in a previous work [27] and is presented in Figure 1. The MIS/NIS deposition on the antenna surface was done by spin coating using a suspension of MIS or NIS in THF, after which the polymer layer was air-dried for 24 h. The spin coating parameters were as follows: speed = 1000 rpm, acceleration = 4000 rpm and time = 40 s. The polymer suspension was prepared as follows: 25 mg of MIS or NIS were transferred in 4 mL of THF containing 8 mg of PVC and were stirred until obtaining a homogenous solution.

The microwave antenna was connected to the vector network analyzer. The antenna was immersed in a beaker containing 100 mL (water/ethanol, 90/10, *v*/*v*) solution, 100 mL of white wine, or 100 mL of red wine, which was considered a blank. After successive addition of increasing volumes of a stock solution of fungicides at 100 mg L^−1^, five measurements were taken for each fungicide concentration. After each addition of the stock solution, the sample was stirred for two minutes with a glass rod and was allowed to stand one minute before taking the measurement.

Before each experiment, the Vector Network Analyzer (VNA) was calibrated. The frequency range varied between 50 MHz and 8 GHz with a span of 0.9 MHz. The microwave input power of the measurement was defined by 0 dBm. The response of the microwave sensor is generated by the VNA and is characterized by the reflection coefficient Γ(f). It is a complex number representing the ratio between the incident wave and the reflected wave and is described by the following equation:

(1) Γ(f)=Re(f)+jIm(f)

Re represents the real part of the coefficient and Im the imaginary part. The exploited signals were obtained from the relative variation of the reflection coefficient during the immersion in a sample, in comparison with the immersion in a reference blank solution. It was calculated from the following equation:

(2) ΔΓΓ=(Γ(sample)−Γ(blank))Γ(blank)

### 2.4. Chemometric Treatments

Transformation and logarithmic scaling was implemented on the Γ(f) coefficient, giving:

(3) A=20log(Re2+Im2)

where *A* represents the amplitude.

It was possible to do the study using the real and imaginary part of the reflection coefficient (Equation (1)). In order to allow the same study to be carried out using VNA or a less expensive device such as an SNA (scalar network analyzer), we chose to use the amplitude in dB.

Raw data (two txt files per sample analysis) were first imported into Excel (Microsoft Office 2016) and merged using Equation (3). Data files were displayed in a matrix format, where rows were observations or samples and columns were variables. All data files were then imported into The Unscrambler^®^ software (v10.5, Camo, Trondheim, Norway) and treated with the same software.

Exploring data, and examining observations and variables one by one, is usually time consuming and often precludes meaningful conclusions. This problem is addressed by using multivariate (multidimensional) analyses, which additionally provide summarizing charts. In general, unsupervised learning such as principal component analysis (PCA) is used to find hidden structures in unlabeled data and seeks to discover natural groupings in the data. PCA was performed on the normalized spectral data (normalization on unit vectors) [30] in order to investigate if the whole signal was useful for discriminating different samples (differences coming from various parameters such as concentration, type of polymer and measurement medium).

PCA allows a reduction of variables and mostly provides projections of data in a new space related to explained variances among the dataset. The new obtained axes are called principal components (PCs) and are constructed with linear combinations of the original variables, in order to keep most of the variance in the first PCs. Here, the data came from spectroscopic analyses. Variables are continuous in nature, so the loadings are not represented in the same way as for discontinuous variables such as physicochemical data. In spectroscopic cases, the values of the loadings of each PC are represented in a plot, where the values of the loadings of component PCs are on the *Y*-axis and the scale corresponding to the experimental unit is on the *X*-axis. Thus, they can be interpreted as a spectrum [31].

Partial least squares regression (PLSR) was also performed to find relations between the microwave signals and concentrations of the compound of interest. PLS is of particular interest because it is able to analyze data with strongly collinear (correlated), noisy and numerous X-variables, and simultaneously to model several response variables Y. PLS generalizes and combines features from principal component analysis and multiple regression.

It is also essential to determine the correct complexity of the model. With numerous and correlated X-variables, there is a substantial risk for over-fitting, i.e., getting a well-fitting model with little or no predictive power. Hence, a strict test of the predictive significance of each PLS component is necessary, followed by stopping when components start to be nonsignificant. Cross-validation is a practical and reliable way to test this predictive significance. It is performed by dividing the data in a number of sets and then by developing a number of parallel models from the reduced data with one of the deleted sets [32].

PLS performances were evaluated based on the random full cross-validation coefficient of determination (R^2^) and root mean square error (RMSE) for calibration and validation groups (RMSEC and RMSEP).

## 3. Results and Discussion

The aim of this study was the use of a microwave sensor covered with a molecularly imprinted polymer layer to monitor three fungicides (iprodione, procymidone and pyrimethanil) in model wine and in white wine. Those three molecules were chosen for their structural homology (chlorobenzyl structure or amino group, see supplementary data Scheme S1) and because of their intensive use in agriculture, especially in the wine industry. Iprodione was chosen as a target fungicide because it is present in most European and world wines. An evaluation carried out by the French Agency for Food Environmental and Occupational Health and Safety [33] has shown that iprodione is an endocrine disruptor. Given its dangerous nature, the use of iprodione was banned in June 2018 according to the European Commission (EU) 2017/2091 regulation concerning the nonrenewal of the approval of this active substance [5]. A study carried out in 2005 by the French Ministry of Agriculture in wine-growing regions in France over 14 years (1990–2003) showed that the active substance with the highest transfer rate from grapes to wine was iprodione [34]. The latter was detected in 100% of the wine samples. Procymidone (93%) and pyrimethanil (85%) also showed a strong presence among wine samples made from contaminated grapes.

The molecularly imprinted silica (MIS) and its control (NIS) were contacted with increasing iprodione concentrations ranging from 10^−5^ to 7·10^−4^ mol/L in a hydro alcoholic solution. Binding isotherms and Scatchard plots are given in the supplementary data (Figure S1). The binding affinity constant of MIS (K = 13.4 mL/µmol) was lower than the corresponding NIS value (19.6 mL/µmol). However, the number of interaction sites was higher for the MIS (94.2 µmol/g) in comparison with the NIS (9.6 µmol/g). The binding kinetic experiment showed that equilibrium was rapidly reached after 20 min.

**Figure 2:**
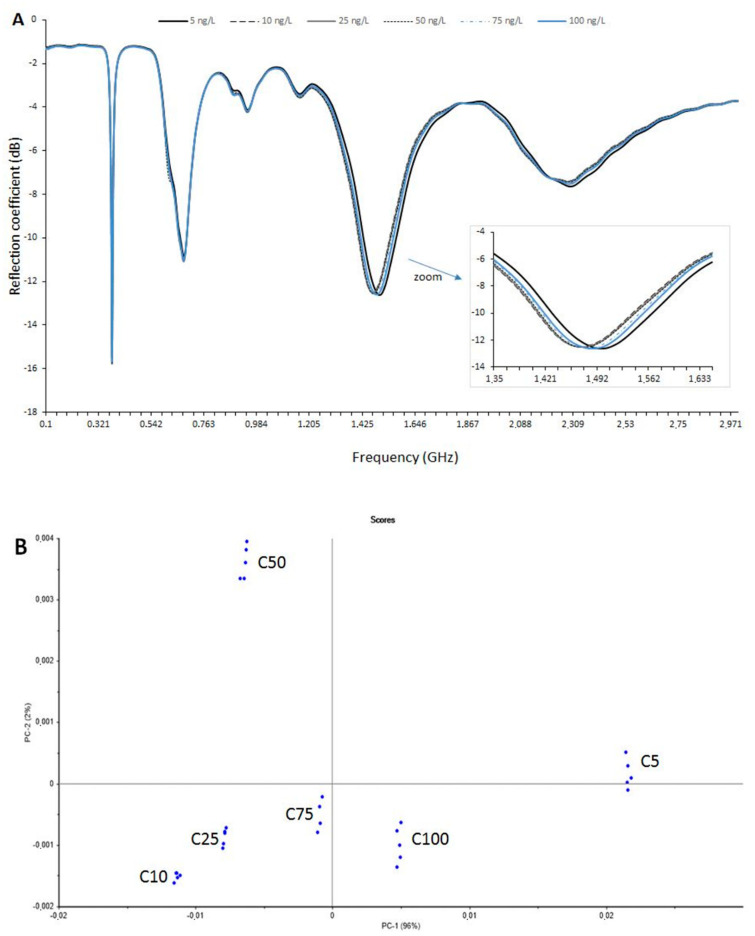

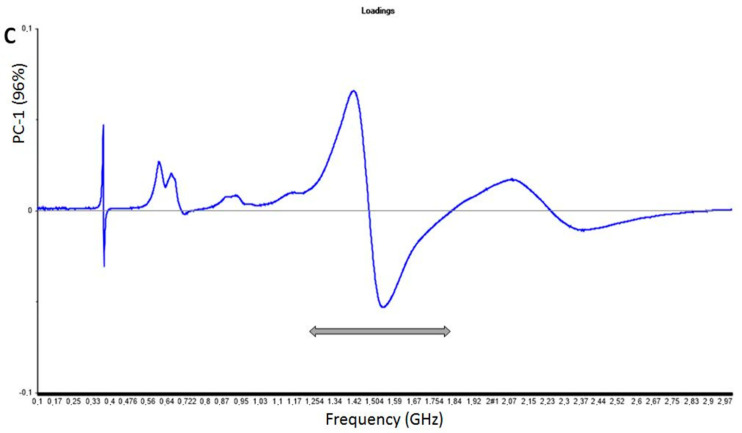
(**A**) Detection of iprodione in model wine medium (water/ethanol, 90/10, *v*/*v*). Representative average microwave spectra (100 MHz–3 GHz) generated by a microwave sensor coupled to MIS. Iprodione concentrations varied from 5 to 100 ng.L^−1^. (**B**) Detection of iprodione in model wine medium (water/ethanol, 90/10, *v*/*v*) using the microwave sensor coupled to MIS. PCA scores plot of 29 iprodione samples. Each spectrum was normalized by unit vector normalization. (**C**) Detection of iprodione in model wine medium (water/ethanol, 90/10, *v*/*v*) using the microwave sensor coupled to MIS. Loading plot for the first principal component (PC1). Characteristic bands are highlighted by a double arrow.

The microwave sensor coupled to MIS was used to detect iprodione in the model wine medium (water/ethanol, 90/10, *v*/*v*). Iprodione concentrations varied from 5 to 100 ng L^−1^. The corresponding microwave spectra are presented in Figure 2A. The slight differences between curves required the use of chemometric tools to analyze the obtained results.

The PCA scores and loadings resulting from the analysis of the iprodione replicates at different concentrations are shown in Figure 2B,C. Each iprodione sample was analyzed five times for each concentration, i.e., 5, 10, 25, 50, 75 and 100 ng L^−1^ (named C5, C10, C25, C50, C75 and C100). At C75, there was one outlier (due to a measurement error), and it was not considered for the multivariate analysis (only four replicates instead of five). Each spectrum was normalized by unit vector normalization. The first two principal components (PC1 and PC2, respectively) accounted for 98% of the total variance in the data.

From the score plot (Figure 2B), it can be seen that all concentrations are well separated and distinguished. The plot also shows that the measurements are repeatable. Differences between C5 and higher concentrations come mainly from the 1.2–1.8 GHz area (Figure 2C). This frequency band corresponds to the strongest contributions of PC-1.

The microwave sensor is able to differentiate solutions at 5 ng L^−1^ from others at higher concentrations. The detection of fungicides in the ng L^−1^ range is very important because it occurs at lower concentrations than the authorized limits in wine (2000 µg L^−1^) and in water (100 ng L^−1^).

In the same way, the detection of the two other fungicides (procymidone and pyrimethanil) was carried out in a model wine medium using the microwave sensor coupled to the MIS. Results similar to those obtained with iprodione were found (supplementary data: Figure S2a,b for procymidone and Figure S3a,b for pyrimethanil). For pyrimethanil, differences between the lower concentration (C5) and higher concentrations (200 and 250 ng L^−1^) come mainly from 1.0–1.3 GHz. For procymidone, the separation between concentrations comes from a larger range of frequencies, between 0.9 and 1.5 GHz (several bands could be distinguished), and the higher contribution is from 0.6 to 0.8 GHz. The part of the target molecule that forms the recognition unit with the monomer corresponds mainly to the carbonyl groups. They interact with the amine group of the monomer through hydrogen bonds. Similarities of interactions are expected for iprodione and procymidone because they have close chemical structures.

**Figure 3:**
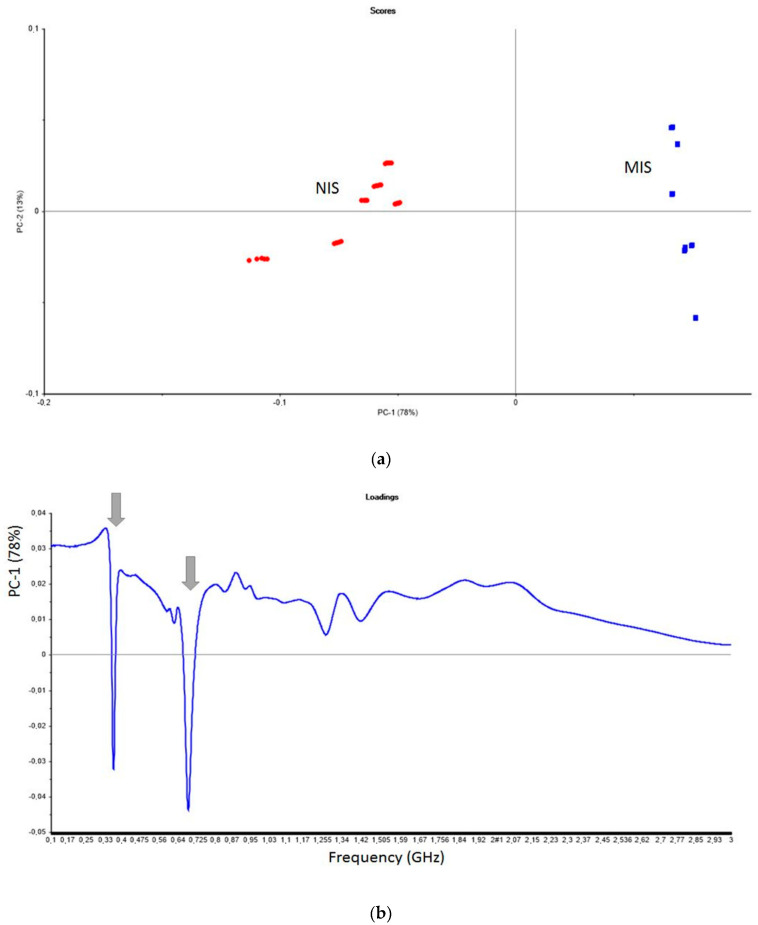
(**a**) PCA model of data presented in the two-dimensional space for the two principal components PC1 and PC2 explaining 91% of the total variance in the data. Example of procymidone detection in model wine medium (water/ethanol, 90/10, *v*/*v*) using the microwave sensor coupled to MIS or NIS. 29 samples from the MIS sensor and 30 samples from the NIS sensor. The procymidone concentration varied from 5 to 250 ng L^−1^. (**b**) Detection of procymidone in model wine medium (water/ethanol, 90/10, *v*/*v*) using the microwave sensor coupled to MIS or NIS. Loading plot for the first principal component (PC1).

In order to study the effect of molecular imprinting, the sensor coupled to the Molecularly Imprinted Silica (MIS) was compared to the sensor coupled to the Nonimprinted Silica (NIS). The PCA of Figure 3a shows a separation along PC1, between MIS and its control NIS sensors, at each concentration of procymidone in the model wine medium. The effect of molecular imprinting is obvious, and the PC2 separates the procymidone concentrations better in the case of the MIS sensor.

The separation between MIS and NIS is mainly due to the 0.4 GHz and 0.7 GHz bands (Figure 3b).

Separations between MIS and NIS are also observed for the two other fungicides, iprodione and pyrimethanil, in the model medium (supplementary data: Figure S4a,b for pyrimethanil and Figure S5a,b for iprodione).

**Figure 4:**
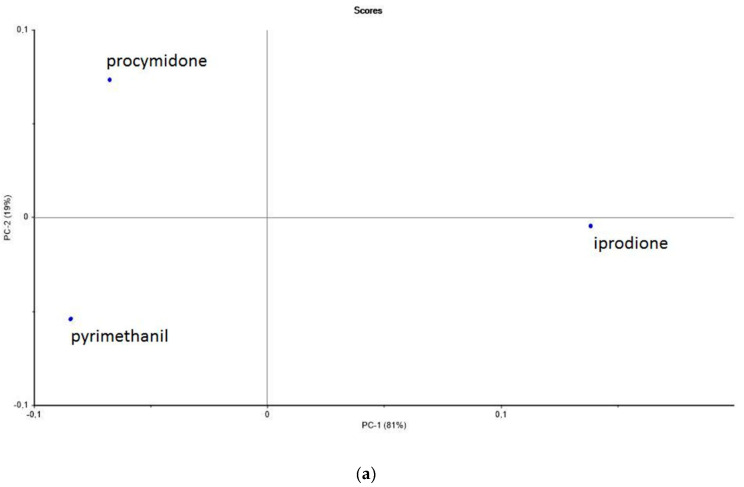

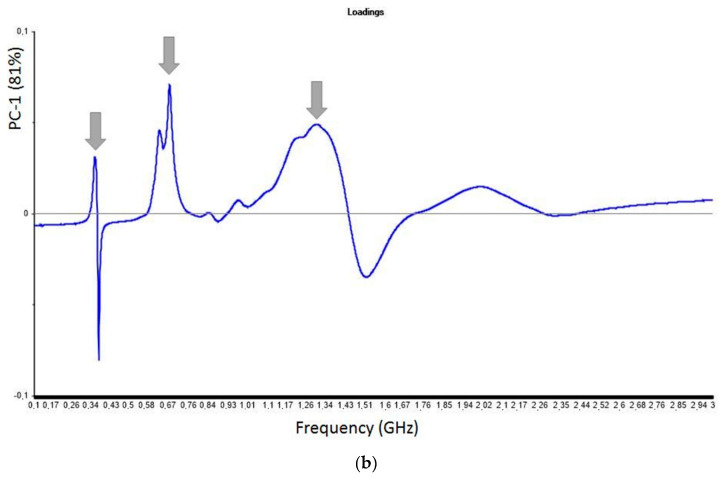
(**a**) PCA scores plot of datasets from the three fungicides at 5 ng L^−1^ analyzed with the microwave sensor coupled to MIS in a model wine medium (water/ethanol, 90/10, *v*/**v**). PC1 and PC2 account for 100% of the total variance in the data. (**b**) Loading plot for the first principal component (PC1) of three fungicides datasets at 5 ng L^−1^ in the model wine medium (water/ethanol, 90/10, *v*/*v*). Spectra were acquired using the microwave sensor coupled to MIS. Characteristic bands are highlighted by arrows.

One important application of the microwave sensor is the simultaneous detection of several fungicides in the same sample. The PCA combining the datasets from the three fungicides (iprodione, procymidone and pyrimethanil) at 5 ng L^−1^ in the model wine medium is presented in Figure 4a. These data were obtained by the microwave sensor coupled to MIS. The first principal component (PC1) was clearly able to separate iprodione from the two other fungicides.

The loadings (Figure 4b) show how the variables (frequencies) are taken into account by the model components. Three bands are observed at definite frequencies. The discrimination between iprodione and the two other fungicides can be attributed to these bands. The discrimination between the three fungicides was also obtained for both concentrations of 50 and 100 ng L^−1^ (see supplementary data: Figure S6a,b for 50 ng L^−1^ and Figure S7a,b for 100 ng L^−1^).

**Figure 5:**
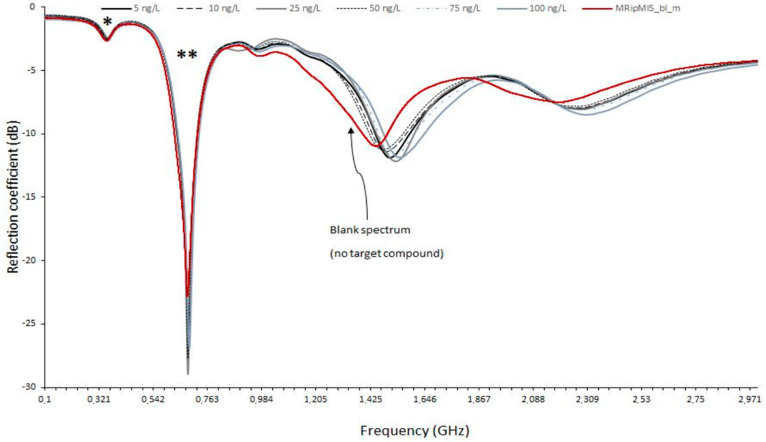
Detection of iprodione in a white wine sample using the microwave sensor coupled to MIS. Representative average microwave spectra. Added iprodione concentrations varied from 5 to 250 ng L^−1^. The two major bands (which contribute mostly to the model) are symbolized by * and **. The red spectrum corresponds to the white wine without any iprodione added.

Once the detection of fungicides in a model wine medium was demonstrated, the microwave sensor was used for the detection of iprodione in a real white wine medium. Figure 5 shows representative microwave spectra of a Chardonnay white wine sample for different added concentrations of iprodione, detected by the microwave sensor coupled to the MIS. For each added concentration, five replicates were measured, and the average spectrum is shown.

**Figure 6:**
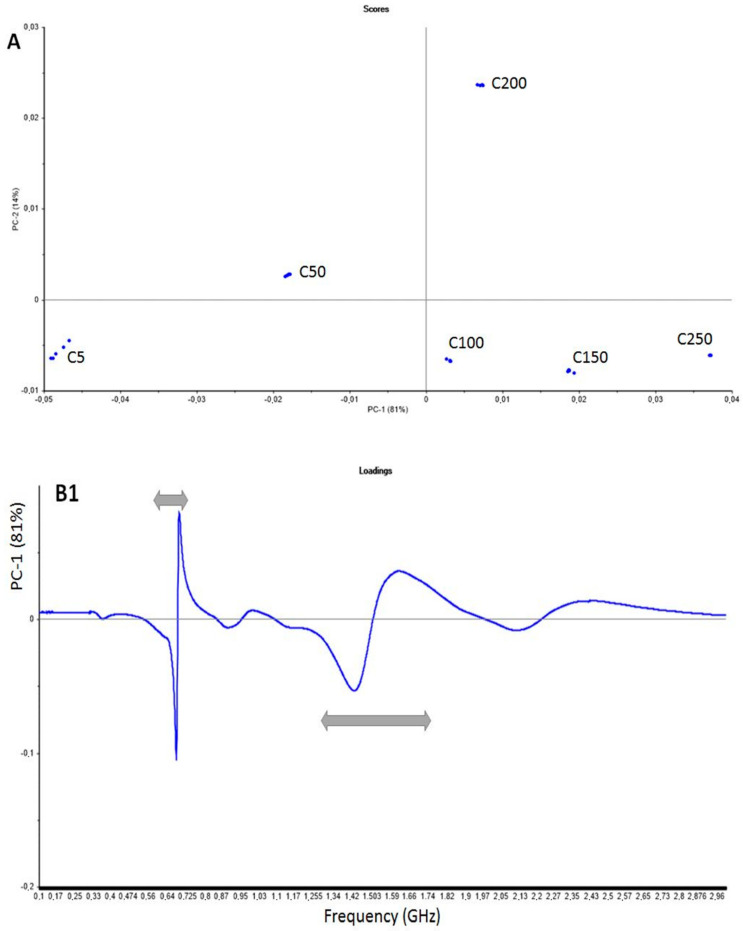

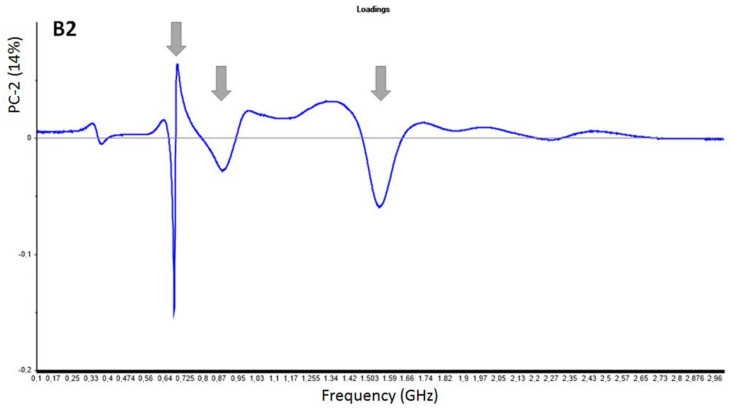
(**A**) Detection of iprodione in a white wine sample using the microwave sensor coupled to MIS. Iprodione concentrations varied from 5 to 250 ng L^−^^1^. PCA scores plot of 30 white wine samples. PC1 and PC2 account for 95% of the total variance in the data. (**B1**) Detection of iprodione in a white wine sample using the microwave sensor coupled to MIS. Loading plot for the first principal component (PC1). Characteristic bands are highlighted by arrows. (**B2**) Detection of iprodione in a white wine sample using the microwave sensor coupled to MIS. Loading plot for the second principal component (PC2). Characteristic bands are highlighted by arrows.

The PCA scores and loadings resulting from the analysis of the white wine replicates for different added concentrations detected by the Molecularly Imprinted Silica (MIS) microwave sensor are shown in Figure 6(A,B1,B2). Each white wine sample was analyzed five times for each added concentration, i.e., 5, 50, 100, 150, 200 and 250 (named C5, C50, C100, C150, C200 and C250). The first two principal components (PC1 and PC2, respectively ng L^−1^) account for 95% of the total variance in the dataset.

From the score plot (Figure 6A), it can be seen that all concentrations were well separated. Differences between samples come mainly from the 0.65–0.85 GHz and 1.3–1.9 GHz areas (Figure 6(B1,B2)) corresponding to the two major bands in the spectrum, symbolized by * and ** in Figure 5. The application of chemometric tools to the microwave spectra of the white wine samples enables the detection of iprodione at very low concentrations, lower than the maximum limit authorized in wine.

A comparison between white wine and model wine media was evaluated for different concentrations of iprodione. PCA scores and loadings for real and model wine samples spiked with iprodione and detected by the microwave sensor coupled to the MIS sensor are shown in the supplementary data (Figure S8a,b). Fungicide concentrations are well separated on the first principal component. Besides the previously identified bands, another band near 6 GHz is responsible for the distinction between the model and real wines.

In sensor applications, it is of primary importance to correlate the sensor signal to the fungicide concentrations. For this reason, PLSR models with one y-variable (fungicide concentration: PLS1) were developed on the spectral data. Signal data was mean-centered and standardized to the unit variance prior to PLS modelling. The vector of the y responses was mean-centered.

**Figure 7:**
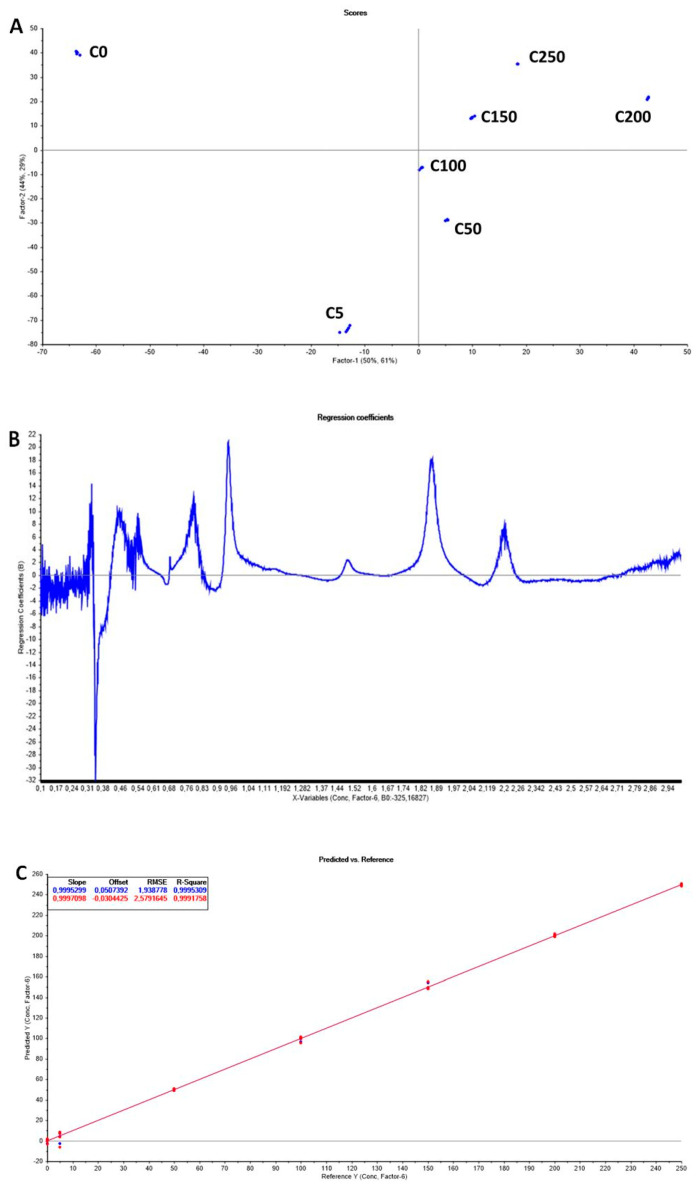
(**A**) Score plot with the first and second PLS components of the PLS regression analysis of iprodione in white wine with the MIS sensor. Iprodione concentrations varied between 0 and 250 ng L^−1^. (**B**) Regression coefficients versus frequency for iprodione in white wine with the MIS sensor (on factor 6). (**C**) Observed and predicted values of iprodione concentrations in white wine using six factors. Coefficient of determination R_C_^2^ for the calibration (blue) and R_V_^2^ for the validation (red) datasets, root mean squared errors (RMSEC for the calibration set and RMSEV for the validation set). Iprodione concentrations varied between 0 and 250 ng L^−1^.

Figure 7A shows the results for iprodione in white wine with the MIS sensor. Concentrations varied between 0 and 250 ng L^−1^.

PLS scores are interpreted the same way as PCA scores. They are the sample coordinates along the model components. The only new feature in PLS is that two different sets of components can be considered, depending on whether one is interested in summarizing the variation in the X- or Y-space.

In Figure 7A, the first two PLS components accounted for 94% (50% (PC1) and 44% (PC2)) of the variability in the microwave spectra (x-variable) and 90% (61% (PC1) and 29% (PC2)) of useful information in the y-variable (concentration of iprodione) in the white wine (MIS sensor) samples contributing to the PLS regression model.

The coefficient of determination (R^2^) and the root mean square error of calibration (RMSEC) are used to evaluate the performance of the model. To assess the feasibility of the calibration model, cross-validation by segmentation (to avoid the influence of replicates) was applied because of the limited number of samples. Moreover, the number of PLSR factors was chosen based on the minimum root mean square error of cross-validation (RMSECV to avoid an over-fitting of the model) [35]. A model was considered satisfactory when it had the higher coefficient of determination (R^2^), the lowest root mean square error of calibration (RMSEC) and validation (RMSEV), and the closer RMSEC and RMSEV with the minimal number of factors [36,37].

The optimum number of latent variables (factors) was found to be 6. Errors are calculated on test/train splits using a cross-validation scheme for the splitting. If the splitting of the data is done correctly, it gives a good estimate on how the model built on the dataset at hand performs for unknown cases. It is characterized by the number called RMSECV (see Table 1). The use of an informative region in a spectrum yields a PLS model with a relatively small value of RMSECV and small PLS dimensionality.

Figure 7B shows the regression coefficients versus the frequency for the factor 6. It can be seen that the signal is noisier. If more than six factors are considered, more noise is introduced and the model will give a poorer prediction.

As in PCA, several peaks and valleys at certain frequencies are more important for the determination of the iprodione concentration in white wine.

In Figure 7C, significant correlation coefficients (R_C_^2^ and R_V_^2^ > 0.99) were found. The results revealed that the MIS sensor was able to discriminate samples at various fungicide concentrations and that PLS models should be appropriate for determining low contents of iprodione in white wine.

A preliminary validation of the developed method was conducted in real samples in order to estimate the limit of detection and the recovery percentages. The order of magnitude of the limit of detection (LOD) of iprodione in red and white wines was estimated using a visual representation of the mean relative error (MRE) increment evolution dependent on an analyte concentration from Oleneva’s study [38].

MRE is calculated by the formula given by Equation (4):

(4) $$\mathrm{MRE}=\frac{\left|M-P\right|}{M}$$

With M being the measured value (taken as actual) and P the predicted value. A plot of the averaged MRE against averaged concentrations is used for the visualization of the measurement system performance for various concentration intervals (shown in Figure S9 in the supplementary material). The LOD of iprodione in white wine can be estimated to be between 20 and 30 ng L^−1^. This value is in agreement with the estimated LODmin (21 ng L^−1^) and LODmax (21.5 ng L^−1^) from equations given by Allegrini et al. [39].

The same procedure was applied to iprodione in red wine. Concentrations varied between 0 and 250 ng L^−1^. The PLS model was less accurate than in white wine (RMSECV was higher and the slope and R^2^ were lower, see Table 1). The LOD value can be graphically estimated to be between 10 and 30 ng L^−1^. This value is on the scale with LOD_min_ (9.6 ng L^−1^) and LOD_max_ (19.9 ng L^−1^) obtained by calculation.

The results obtained from the PLS models for iprodione detected by the MIS sensor in white and red wine are summarized in Table 1 and Table 2a,b:

In red wine, the PLS model is less efficient than in white wine, especially at low concentrations, and this is likely due to matrix effects related to the high polyphenol content (see the supplementary data: Figure S10a–c). The LOD was estimated using the graphical method only. It has to be mentioned that the concentration ranges near the LOD are limited. A perspective to enhance the estimation is to increase the concentration levels from 5 to 30 ng L^−1^.

For concentrations higher than the LOD, recovery percentages ranged between 90 and 110% for white wine and between 99 and 100% for red wine. These values are acceptable for trace analyses.

## 4. Conclusions

Microwave sensors coupled to a molecularly imprinted silica (MIS) and its control (Nonimprinted Silica, NIS) were used to monitor three of the most common fungicides in French wines (iprodione, procymidone and pyrimethanil). Iprodione was detected by the MIS sensor in a model wine medium and in a Chardonnay white wine from Burgundy. The developed sensors were able to differentiate hydroalcoholic solutions for different added concentrations of the three fungicides at low levels of concentrations (ng L^−1^). The imprinting effect was demonstrated since the MIS sensor reacted differently from the NIS sensor and separated the fungicides samples having different concentrations better than the NIS sensor did. The developed MIS sensor was able to simultaneously detect all three fungicides at concentrations down to 5 ng L^−1^ in a hydroalcoholic medium and to detect iprodione at concentrations as low as 20 ng L^−1^ in white wine and 10 ng L^−1^ in red wine.

The chemometric PCA and PLS methods were successfully used as exploratory methods for the statistical analysis of microwave spectra. They demonstrated that the whole microwave spectrum was useful for discriminating samples containing different fungicides at varying concentrations when both MIS and NIS microwave sensors generated the data. PLS models applied to the microwave spectra were able to monitor low concentration of iprodione in white and red wines.

## Figures and Tables

**Table 1:** PLS results and estimation of LOD for iprodione using the MIS sensor in white and red wines.

	PLS Model Characteristics			LOD Estimation (ng L^−1^)
	Slope	R^2^	RMSECV	
White wine MIS	0.9997	0.9996	2.6	~20–30
Red wine MIS	0.9574	0.9940	18.7	~10–30

**Table 2:** (**a**) Predicted vs. theoretical concentrations and recovery for iprodione using the MIS sensor in white wine. (**b**) Predicted vs. theoretical concentrations and recovery for iprodione using the MIS sensor in red wine.

(**a**)			
**Conc. Theoretical (ng L^−1^)**	**Conc. Predicted (Mean) (ng L^−1^)**	**Conc. Predicted (sd) (ng L^−1^)**	**Recovery (%)**
5	3.40	1.12	68
50	54.83	1.03	110
100	89.82	0.10	90
150	163.50	0.51	109
200	199.01	0.30	100
250	244.45	0.66	98
(**b**)			
**Conc. Theoretical (ng L^−1^)**	**Conc. Predicted (Mean) (ng L^−1^)**	**Conc. Predicted (sd) (ng L^−1^)**	**Recovery (%)**
5	7.32	6.72	146
10	7.22	4.54	72
25	30.36	6.76	121
50	49.79	13.71	100
100	100.10	9.87	100
250	247.75	6.82	99

## Supplementary Materials

The following figures and tables are available online at https://www.mdpi.com/1424-8220/20/21/6224/s1.
