# Supplementary material for: A First Tentative for Simultaneous Detection of Fungicides in Model and Real Wines by Microwave Sensor Coupled to Molecularly Imprinted Sol-Gel Polymers
Source: Sensors (Basel). 2020 Oct 31;20(21):6224. doi: 10.3390/s20216224 (PMC7662697; doi:10.3390/s20216224)
Supplement: Supplementary file 1 [file sensors-20-06224-s001.pdf]

Supplementary data

A first tentative for simultaneous detection of fungicides in model and real wines by microwave sensor coupled to molecularly imprinted sol-gel polymers

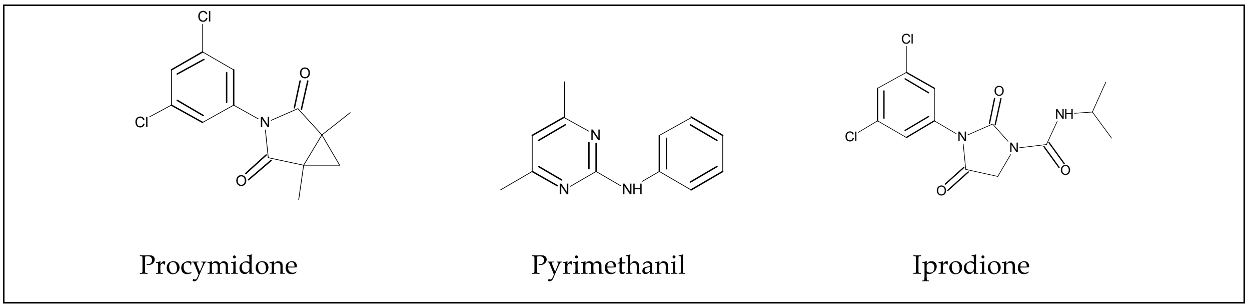

Scheme S1. Chemical structures of the 3 fungicides: Iprodione, pyrimethanil and procymidone

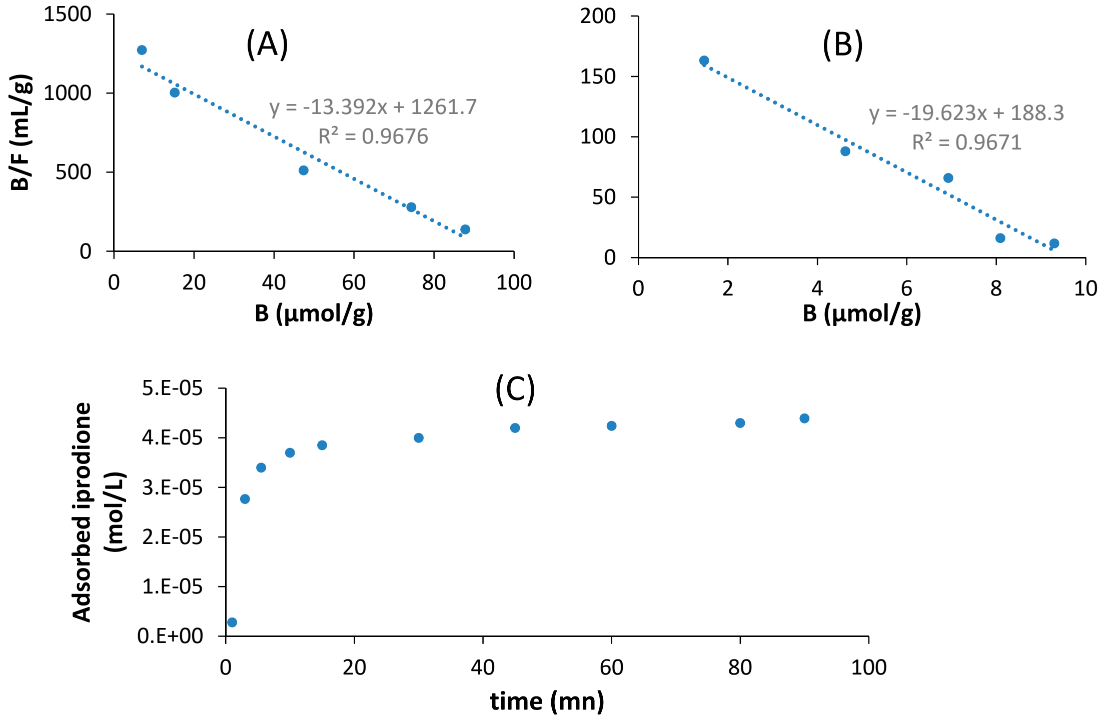

Figure S1. Scatchard plots for iprodione-MIP (A) and iprodione-NIP (B). B is the adsorbed iprodione concentration and F the free one. Binding experiments were performed in (ethanol/water, 50/50, v/v). Iprodione concentrations ranged from 10^{-5} to 7.10^{-4} mol/L. (C) Kinetic adsorption of iprodione on MIP in (ethanol/water, 50/50, v/v) at room temperature. [Iprodione] = 7.10^{-4} mol/L.

Procymidone

Each procymidone sample was analysed 5 times at 5, 50, 100, 150, 200 and 250 ng.L^{-1}.

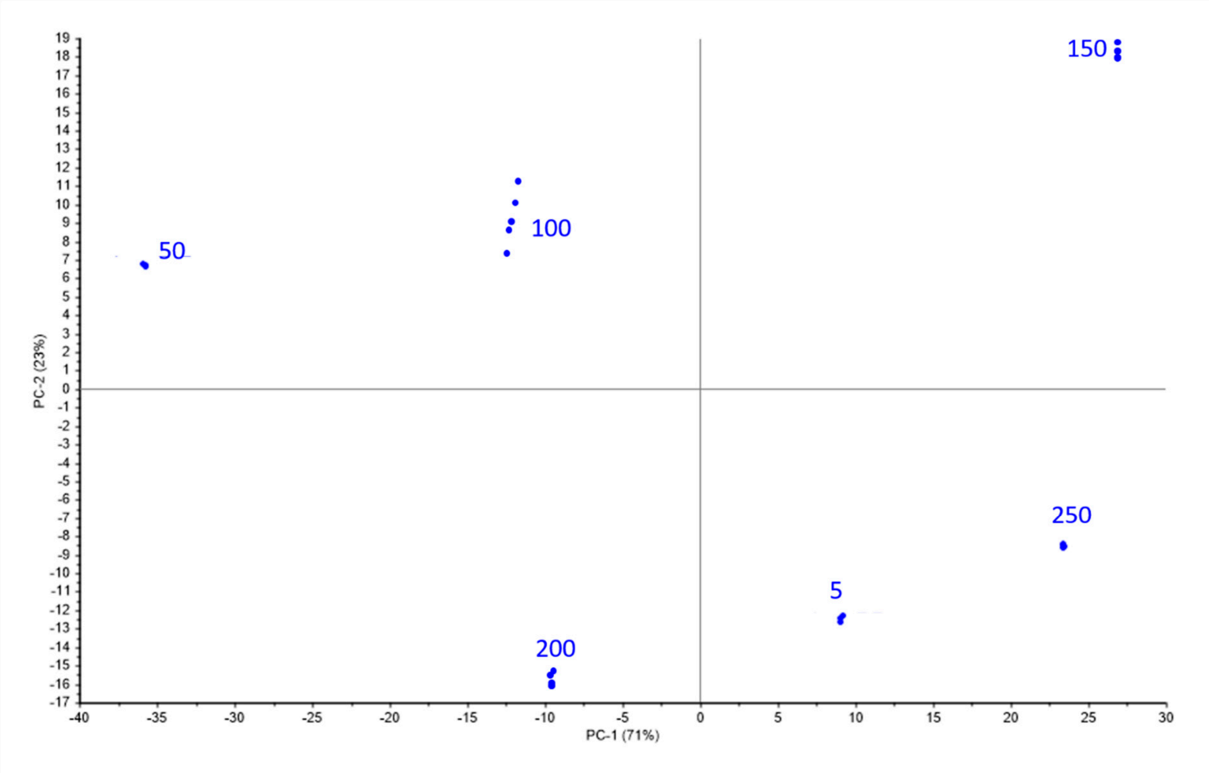

Figure S2a. Detection of Procymidone in model wine medium (water/ethanol, 90/10, v/v) using the microwave sensor coupled to MIS. PCA scores plot of 29 procymidone samples. Each spectrum was normalised by unit vector normalisation.

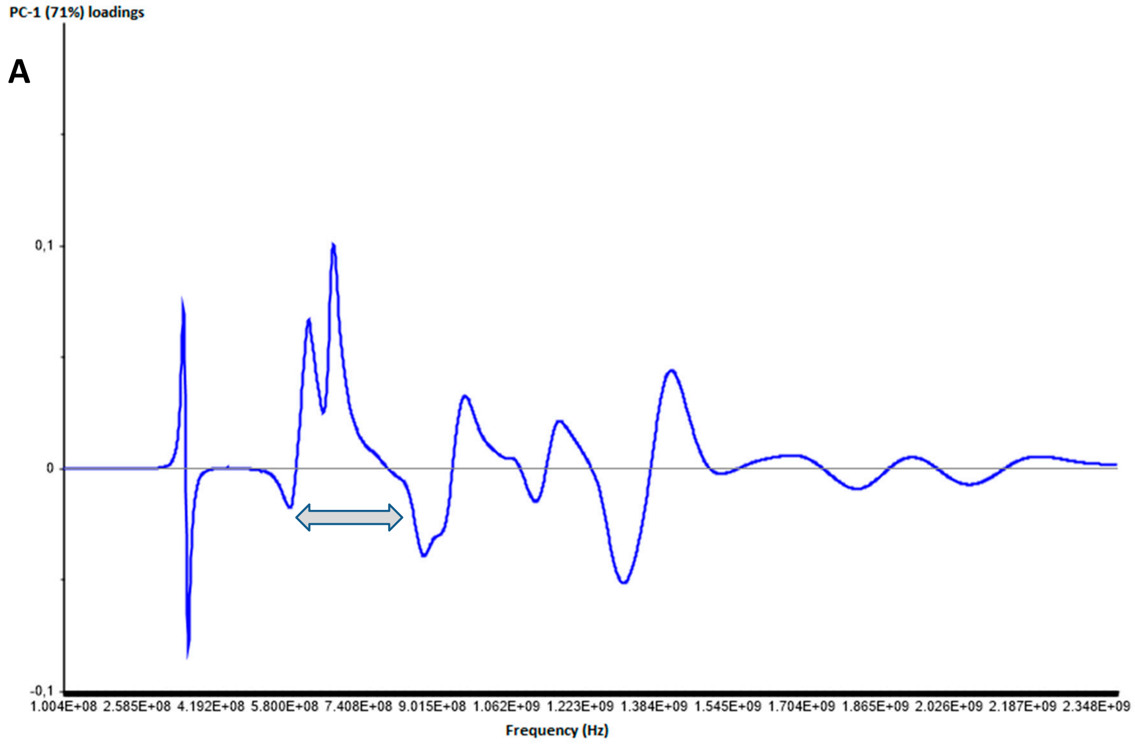

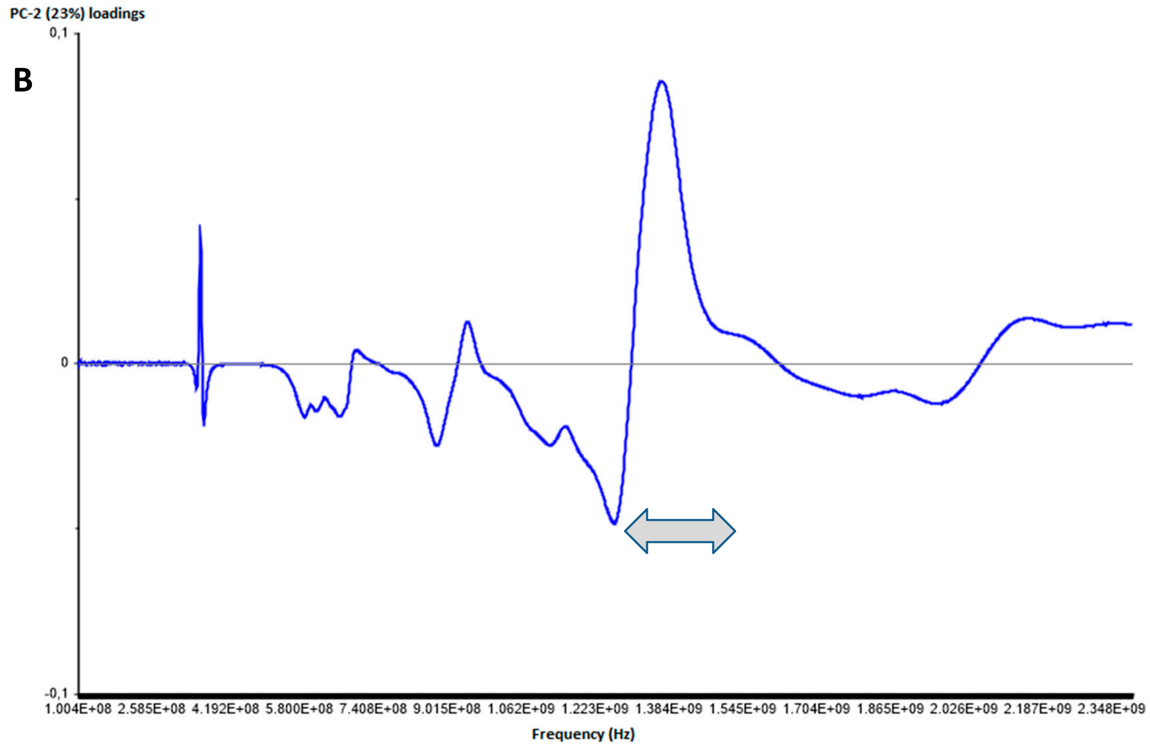

Figure S2b. Detection of Procymidone in model wine medium (water/ethanol, 90/10, v/v) using the microwave sensor coupled to MIS. Loading plot for (A) the first principal component (PC1) and (B) the second principal component (PC2).

Pyrimethanil

Each pyrimethanil sample was analysed 5 times at 5, 50, 100, 150, 200 and 250 ng.L^{-1}.

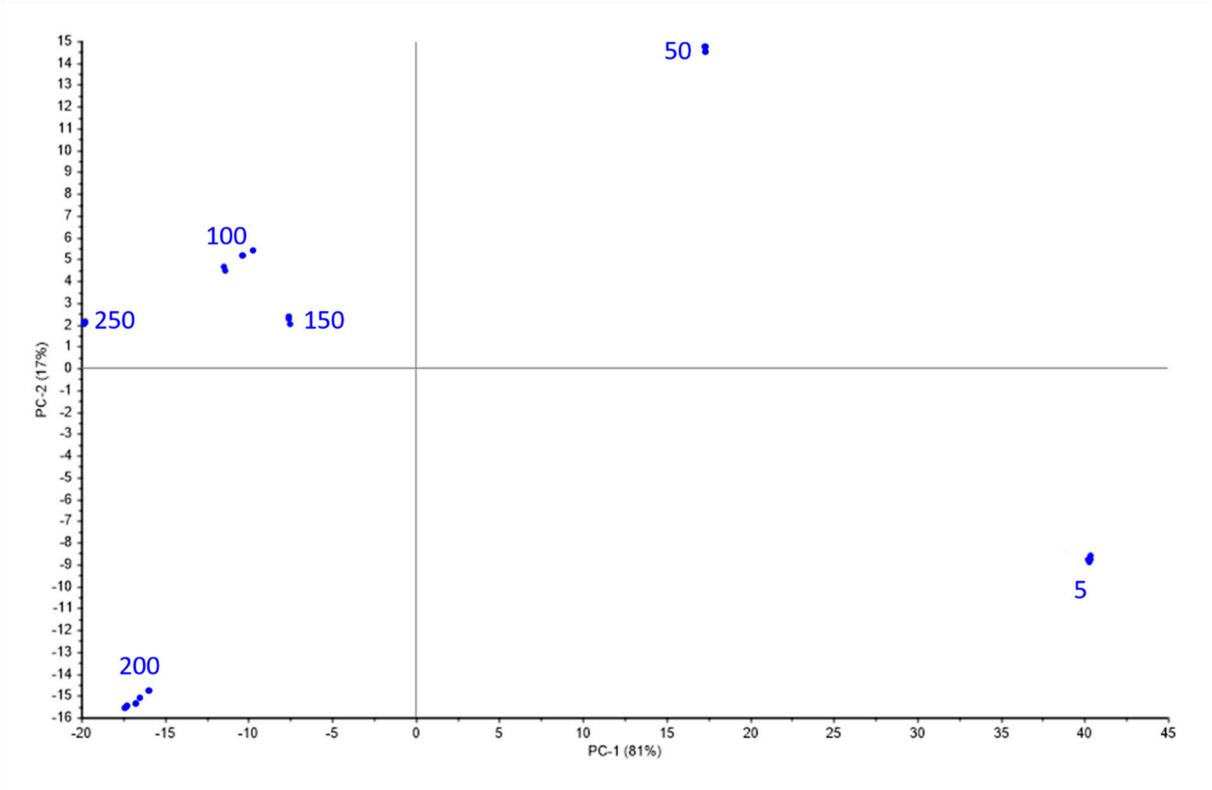

Figure S3a. Detection of Pyrimethanil in model wine medium (water/ethanol, 90/10, v/v) using the microwave sensor coupled to MIS. PCA scores plot of 30 pyrimethanil samples. Each spectrum was normalised by unit vector normalisation.

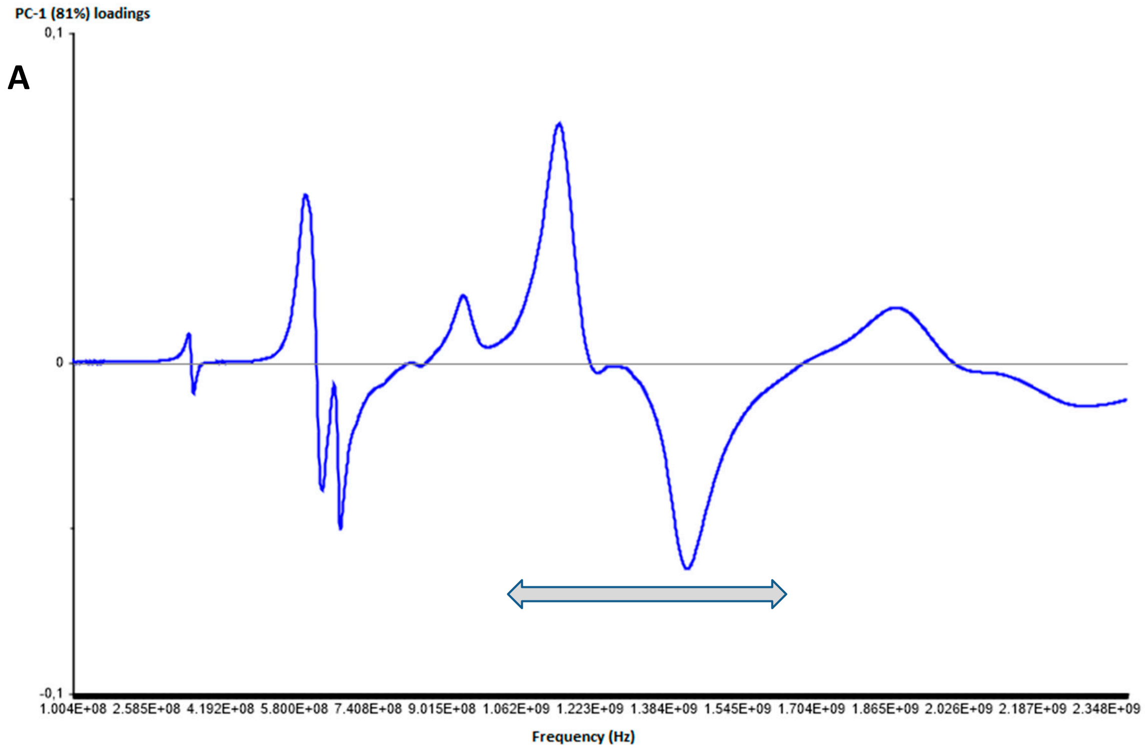

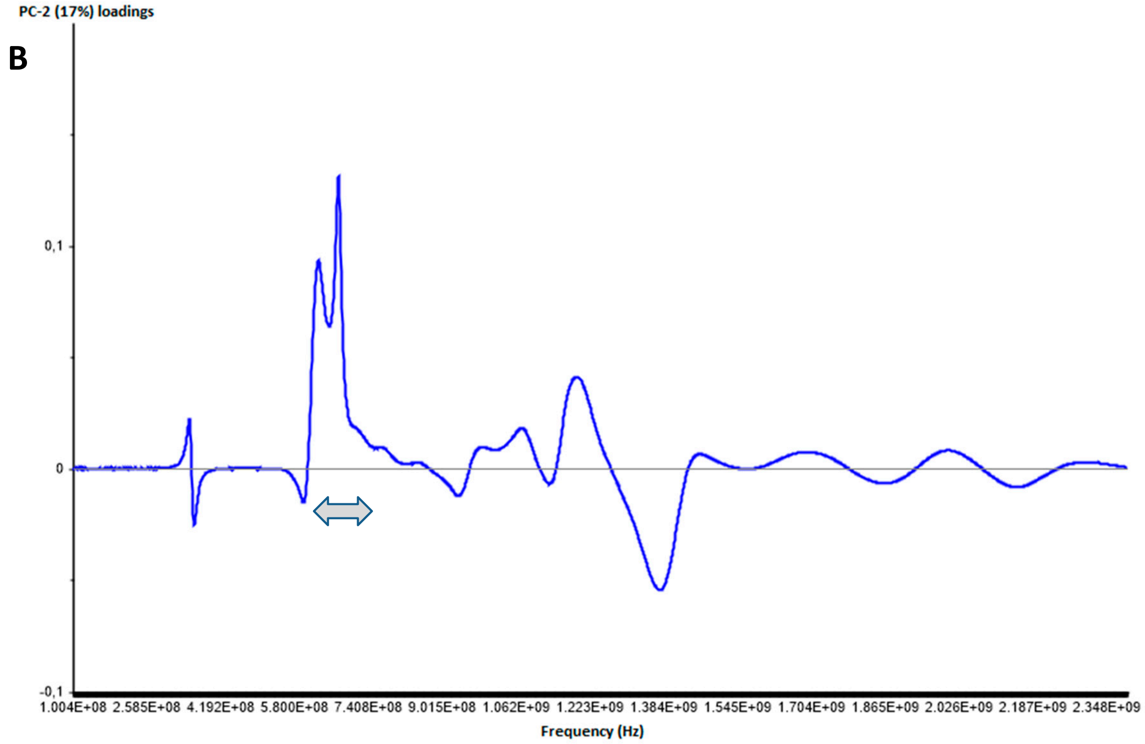

Figure S3b. Detection of Pyrimethanil in model wine medium (water/ethanol, 90/10, v/v) using the microwave sensor coupled to MIS. Loading plot for (A) the first principal component (PC1) and (B) the second principal component (PC2).

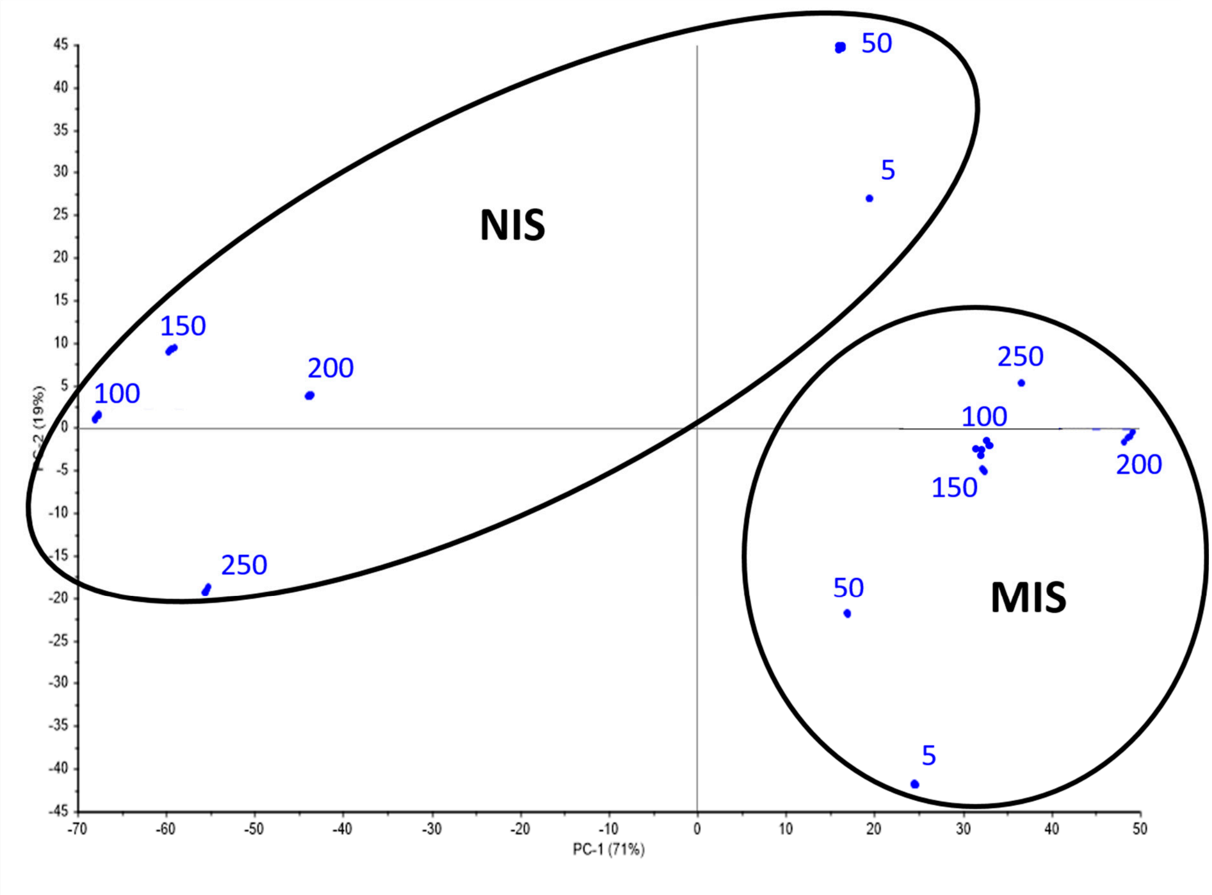

Figure S4a. PCA model of data presented in the 2-dimensional space for the 2 principal components PC1 and PC2 explaining 90% of the information of the data set. Example of pyrimethanil detection in model wine medium (water/ethanol, 90/10, v/v) using the microwave sensor coupled to MIS or NIS. 30 samples from MIS sensor and 30 samples from NIS sensor. Pyrimethanil concentration varies from 5 to 250 ng.L^{-1}.

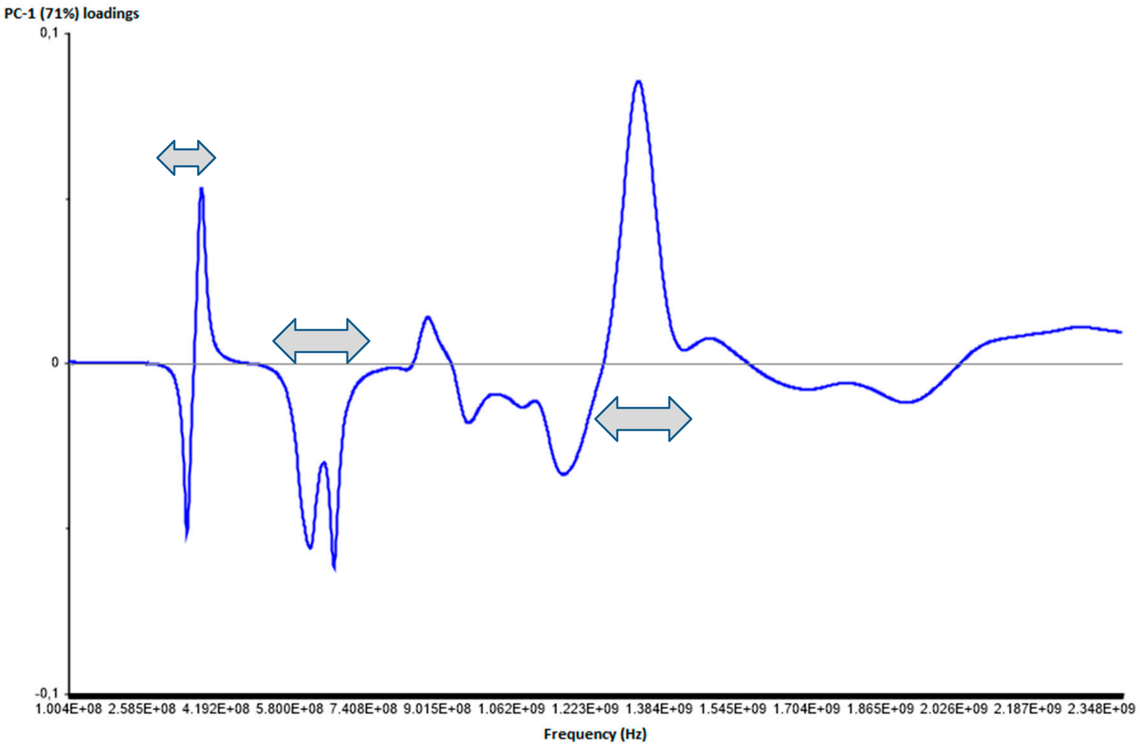

Figure S4b. Detection of Pyrimethanil in model wine medium (water/ethanol, 90/10, v/v) using the microwave sensor coupled to MIS or NIS. Loading plot for the first principal component (PC1).

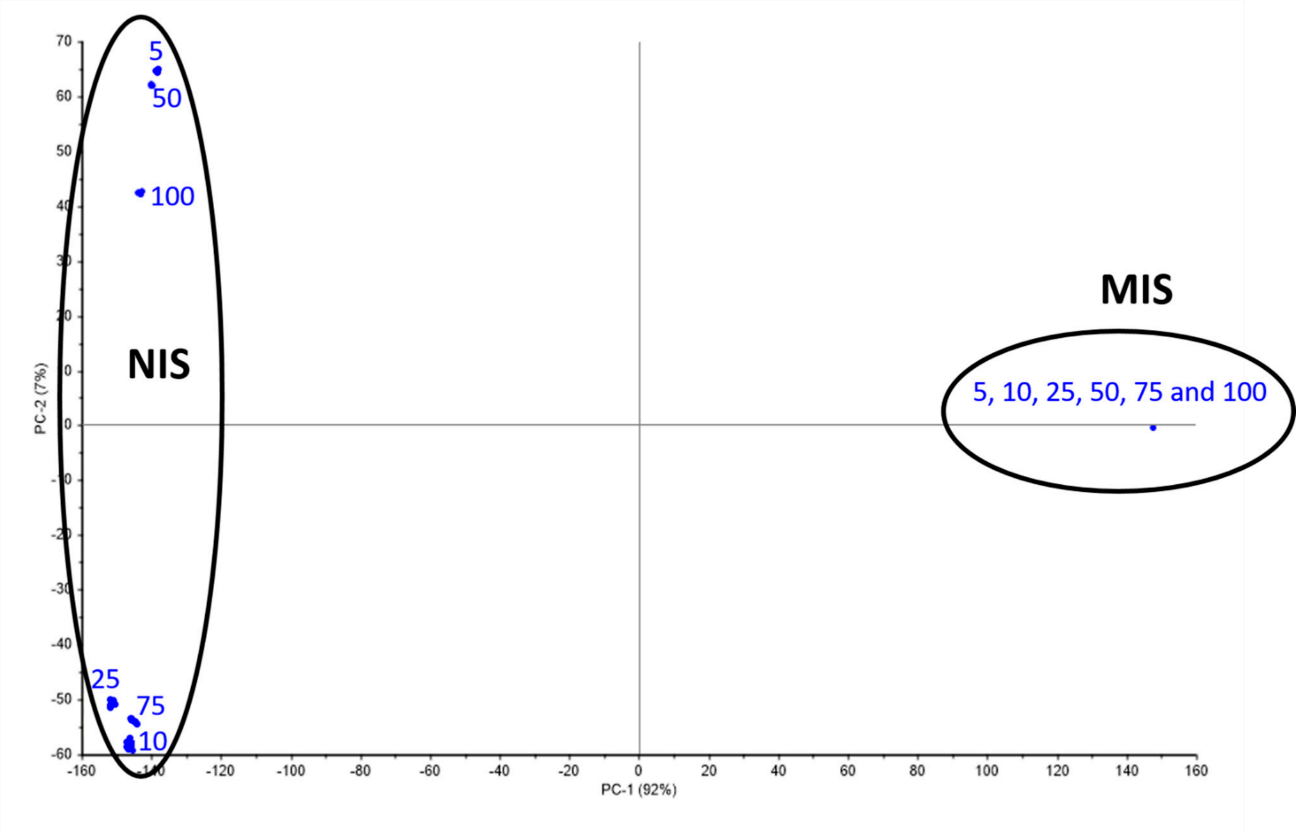

Figure S5a. PCA model of data presented in the 2-dimensional space for the 2 principal components PC1 and PC2 explaining 90% of the information of the data set. Example of iprodione detection in model wine medium (water/ethanol, 90/10, v/v) using the microwave sensor coupled to MIS or NIS. 29 samples from MIS sensor and 30 samples from NIS sensor. Iprodione concentration varies from 5 to 100 $ng.L^{-1}$.

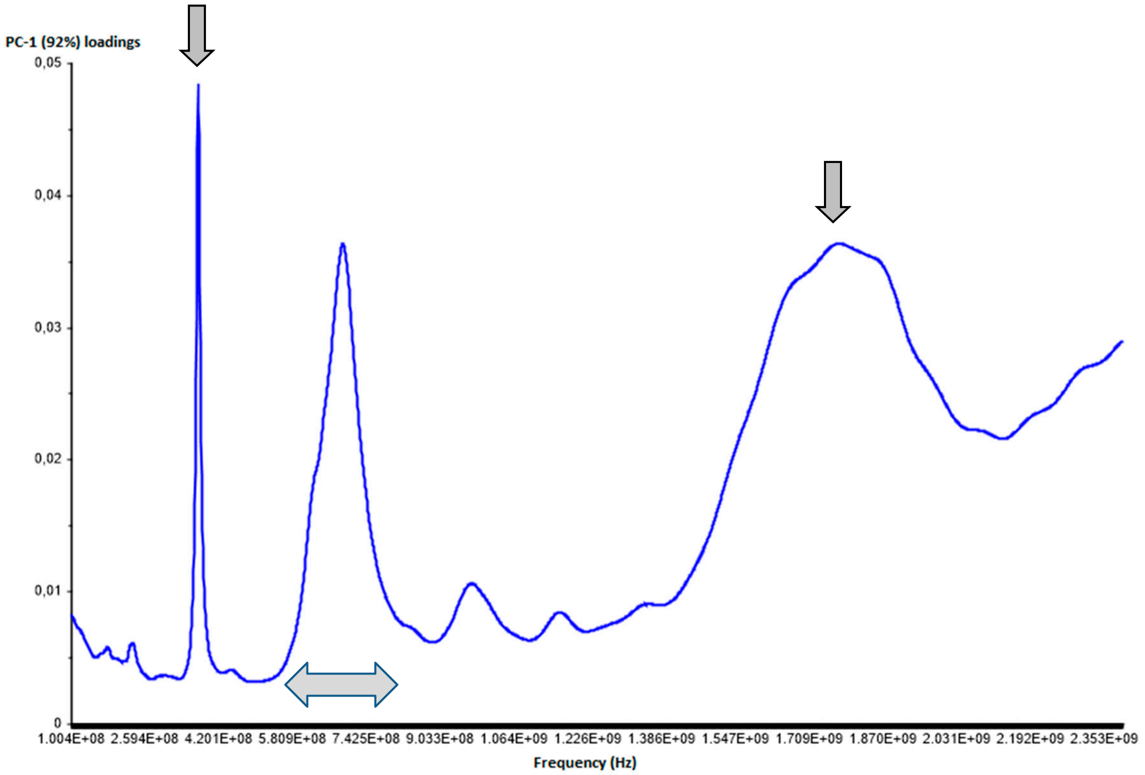

Figure S5b. Detection of Iprodione in model wine medium (water/ethanol, 90/10, v/v) using the microwave sensor coupled to MIS or NIS. Loading plot for the first principal component (PC1).

Iprodione + Pyrimethanil + procymidone

Every samples from different components were analysed 5 times at 50 and 100 ng.L^{-1}.

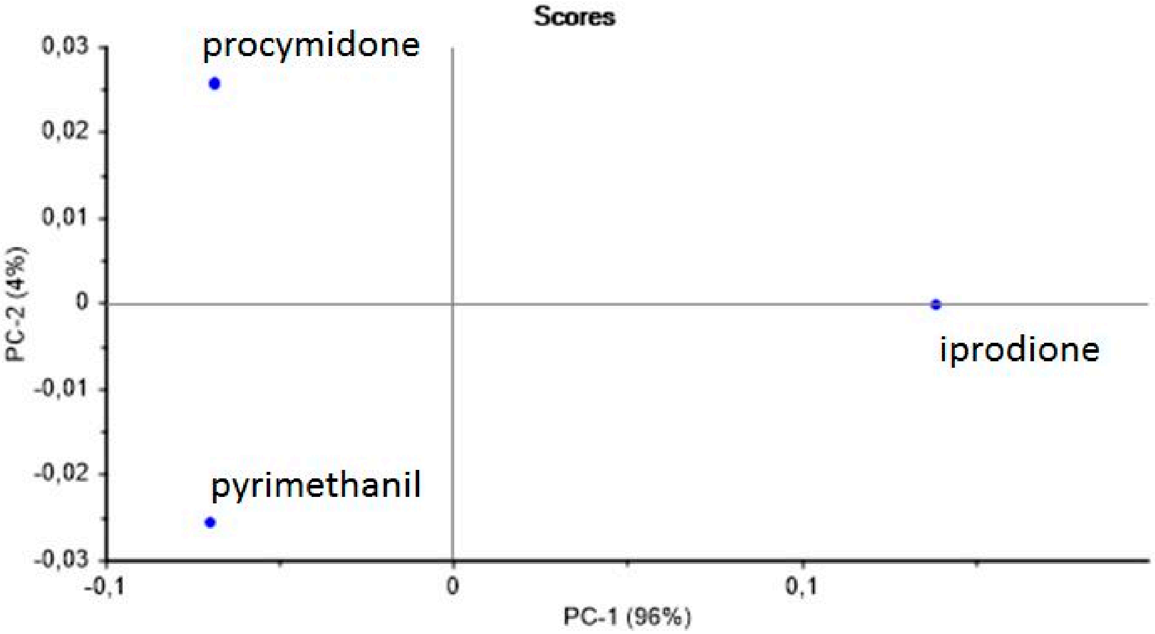

Figure S6a. PCA scores plot of datasets from the 3 fungicides at 50 ng.L^{-1} analysed with the microwave sensor coupled to MIS in a model wine medium (water/ethanol, 90/10, v/v). PC1 and PC2 account for 100% of the total variance in the data.

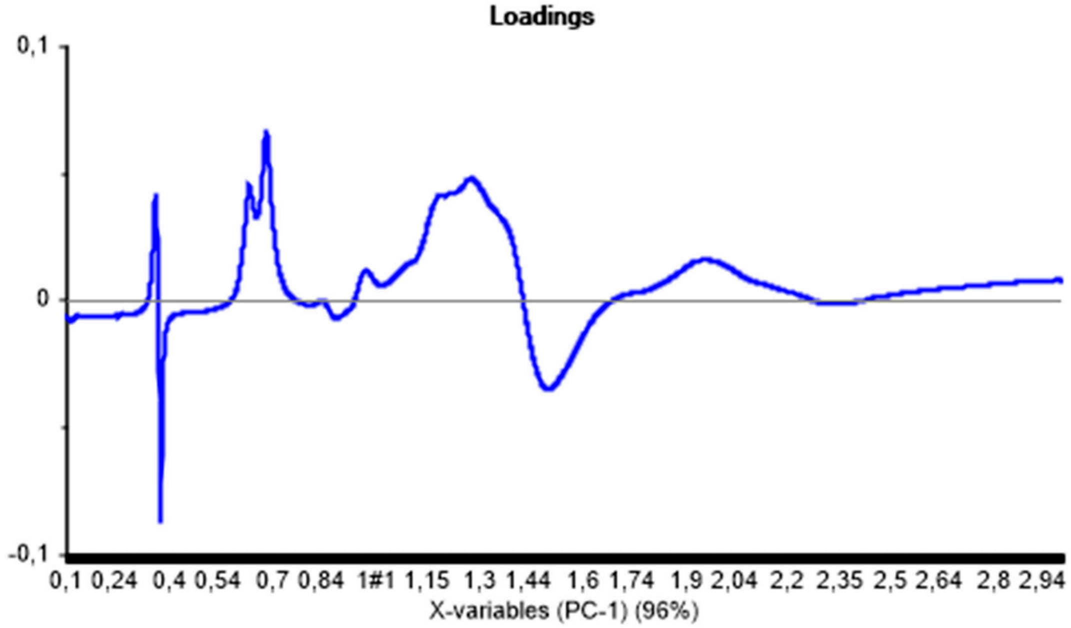

Figure S6b. Loading plot for the first principal component (PC1) of 3 fungicides datasets at 50 ng.L^{-1} in model wine medium (water/ethanol, 90/10, v/v). Spectra were acquired using the microwave sensor coupled to MIS.

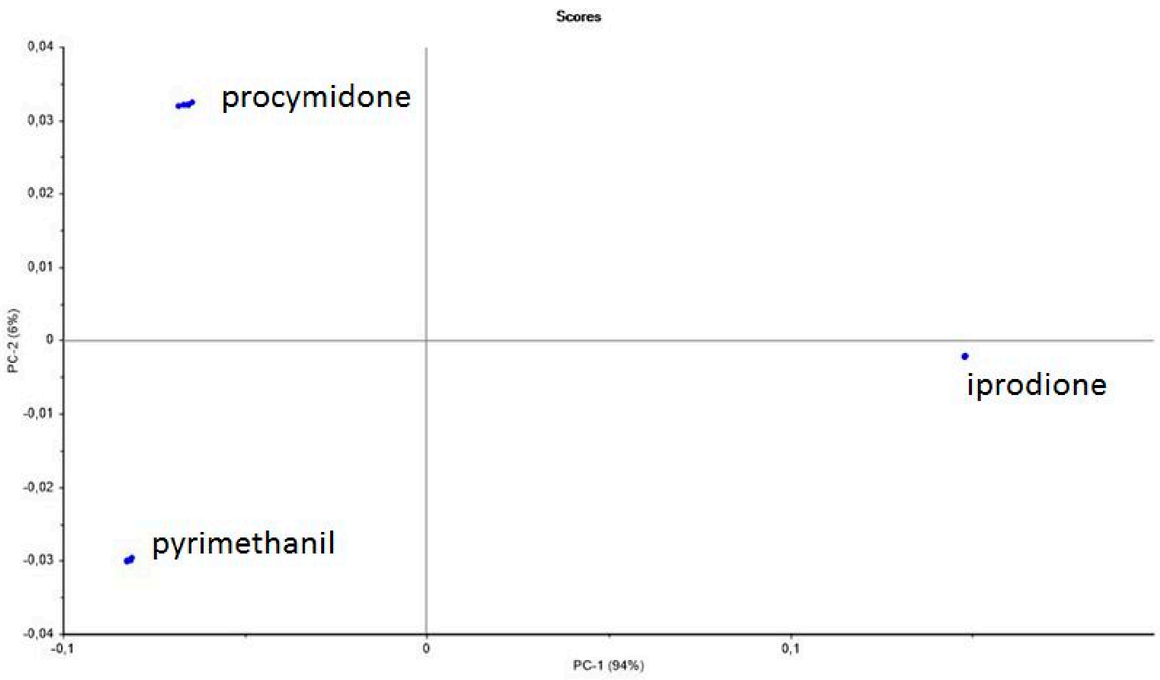

Figure S7a. PCA scores plot of datasets from the 3 fungicides at 100 ng.L^{-1} analysed with the microwave sensor coupled to MIS in a model wine medium (water/ethanol, 90/10, v/v). PC1 and PC2 account for 100% of the total variance in the data.

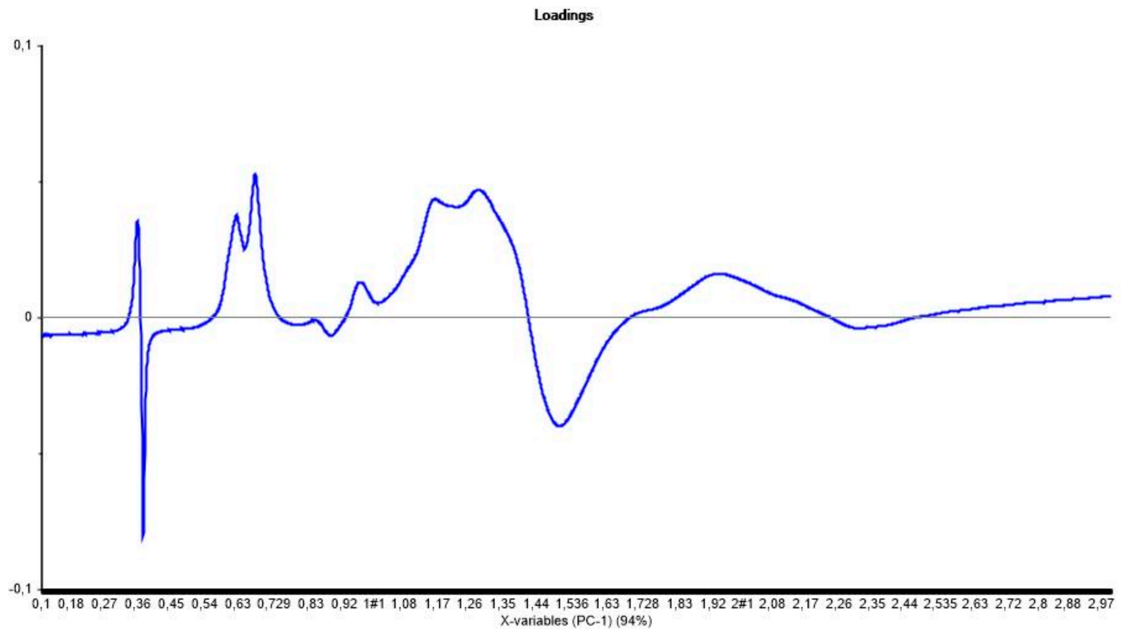

Figure S7b. Loading plot for the first principal component (PC1) of 3 fungicides datasets at 100 ng.L^{-1} in model wine medium (water/ethanol, 90/10, v/v). Spectra were acquired using the microwave sensor coupled to MIS.

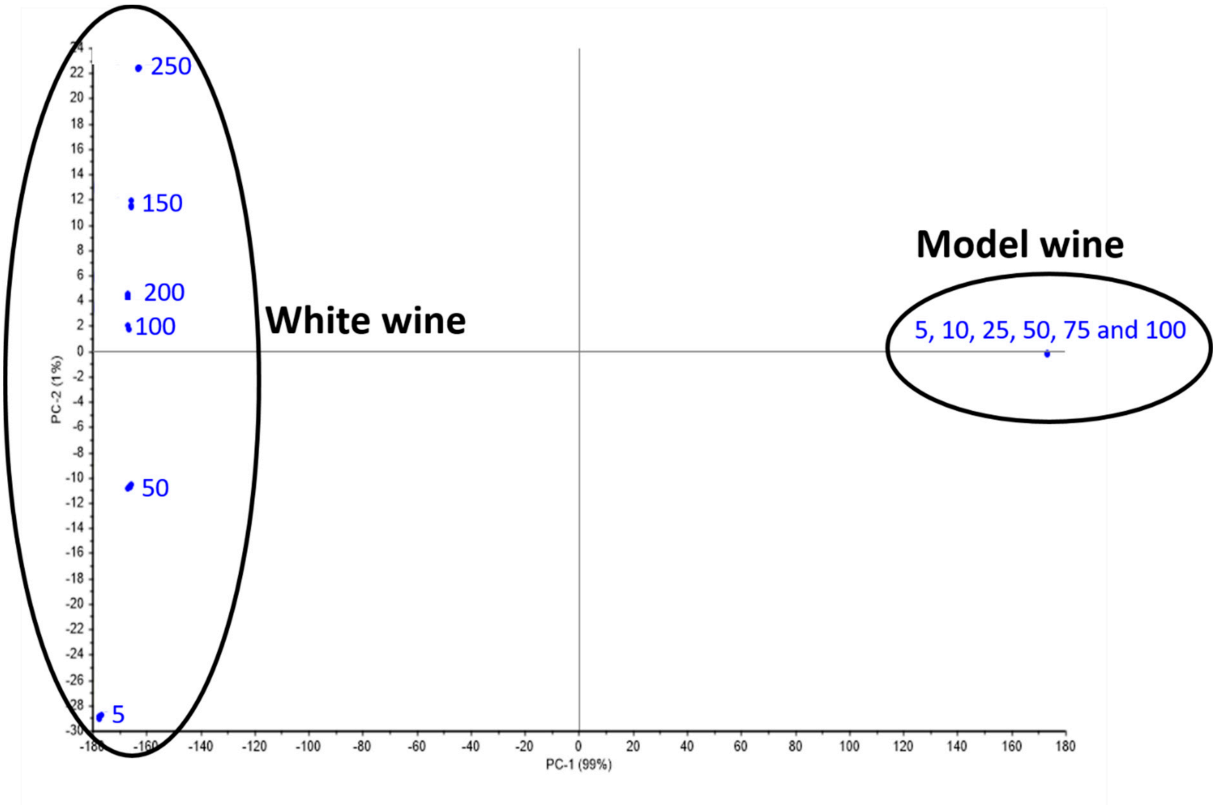

Figure S8a. PCA model of data presented in the 2-dimensional space for the 2 principal components PC1 and PC2 explaining all of the information of the data set. Example of iprodione detection in model wine medium (water/ethanol, 90/10, v/v) and in white wine medium using the microwave sensor coupled to MIS. 29 samples from model wine medium and 29 samples from white wine medium. Iprodione concentration varies from 5 to 100 $ng.L^{-1}$ for model wine medium and from 5 to 250 $ng.L^{-1}$ for white wine model.

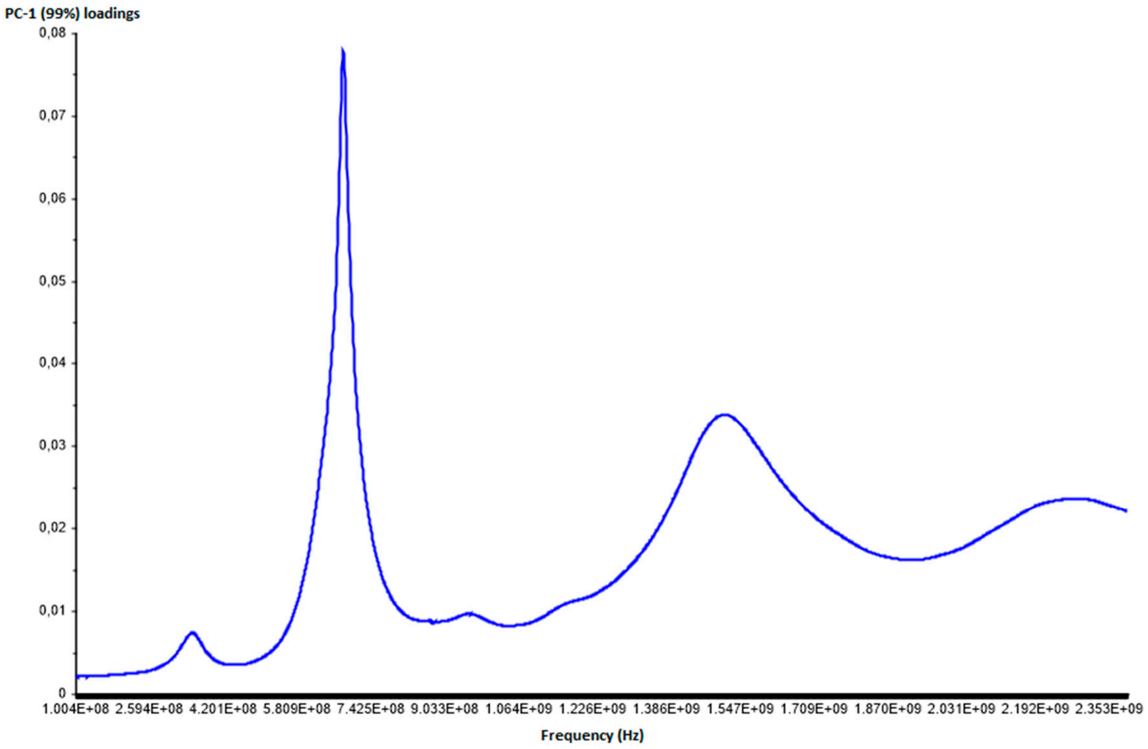

Figure S8b. Detection of Iprodione in model wine medium (water/ethanol, 90/10, v/v) and white wine model using the microwave sensor coupled to MIS. Loading plot for the first principal component (PC1).

PLS analysis for iprodione in white wine, estimation of LOD

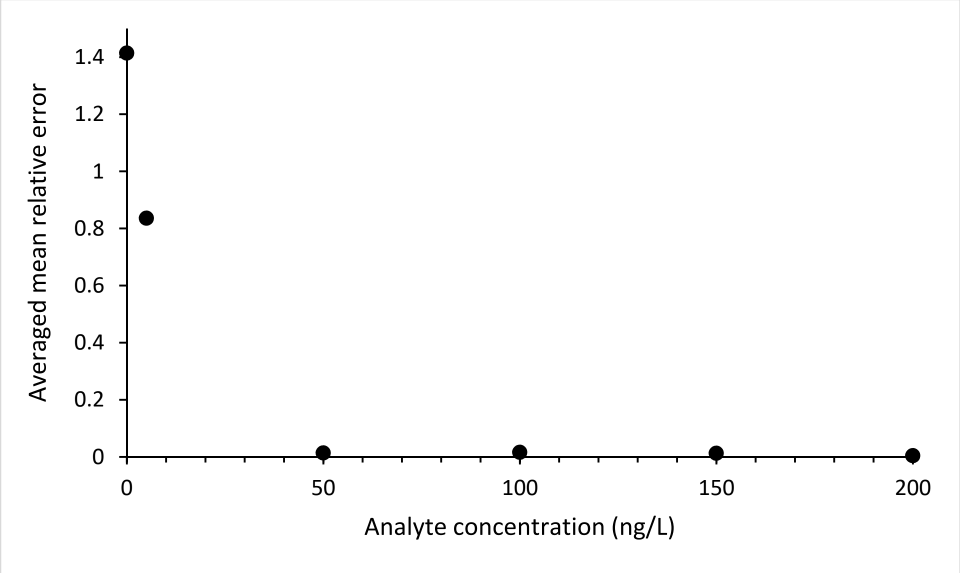

Figure S9. Mean relative error (MRE calculated from equation 4) evolution as a function of concentration for iprodione in white wine. The abrupt decrease of average MRE increment can be observed. Procedure used to draw the curve comes from "Oleneva, E.; Khaydukova, M.; Ashina, J.; Yaroshenko, I.; Jahatspanian, I.; Legin, A.; Kirsanov, D. A Simple Procedure to Assess Limit of Detection for Multisensor Systems. Sensors 2019, 19, 1359."

Calculation of multivariate LOD comes from Allegrini, F.; Olivieri, A. IUPAC-Consistent Approach to the Limit of Detection in Partial Least-Squares Calibration. Anal. Chem. 2014, 86, 7858–7866:

$$LOD_{min} = 3.3\sqrt{[SEN^{-2}var(x)(1 + h_{0min}) + h_{0min}var(y_{cal})]} \quad (1)$$

$$LOD_{max} = 3.3\sqrt{[SEN^{-2}var(x)(1 + h_{0max}) + h_{0min}var(y_{cal})]} \quad (2)$$

Where var(x) is the variance in instrumental signals, h is the sample leverage, and var(ycal) the variance in the calibration concentrations, SEN is the sensitivity given in PLS by the inverse of the length of the regression coefficients.

Results for white wine:

var(x) [(ng/L)^2]	var(ycal))	h_{0min}	h_{0max}	SEN
0.0003	5.105	0.176	0.229	0.003

PLS analysis for iprodione in red wine

Each iprodione sample was analysed 5 times at 0, 5, 10, 25, 50, 100 and 250 ng.L^{-1}.

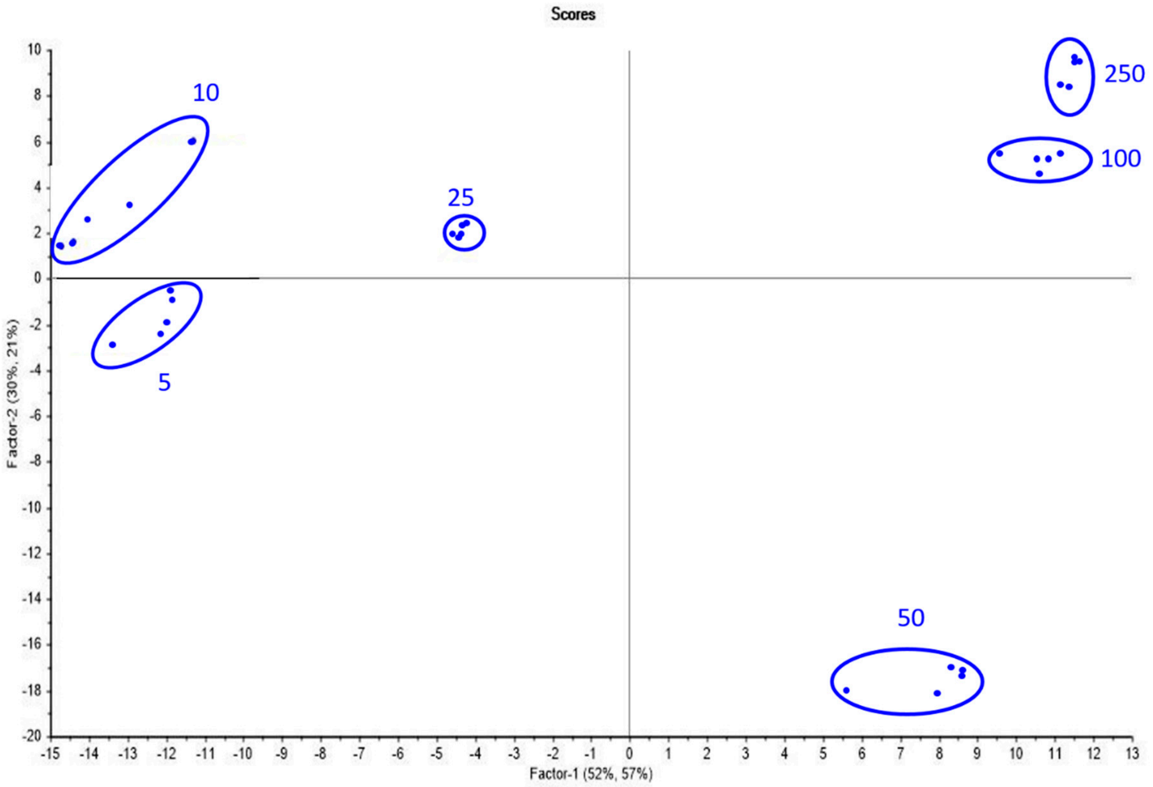

Figure S10a. Score plot with first and second PLS components of the PLS regression analysis of iprodione in red wine with the MIS sensor. Iprodione concentrations varied between 0 and 250 ng.L^{-1}.

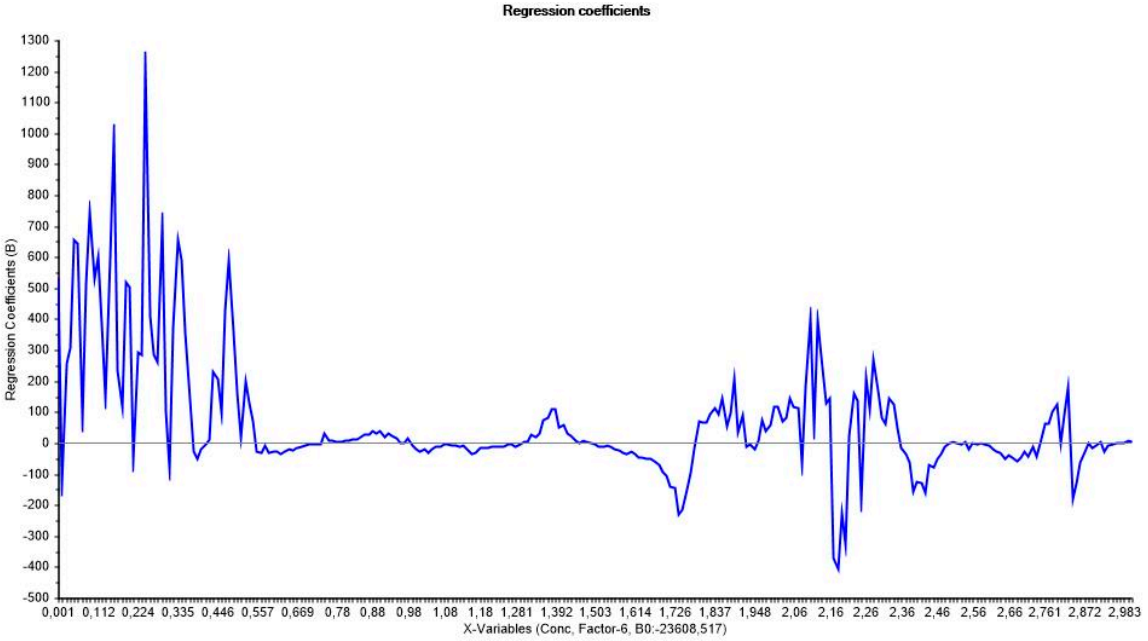

Figure S10b. Regression coefficients versus frequency for iprodione in red wine with MIS sensor (on factor 6).

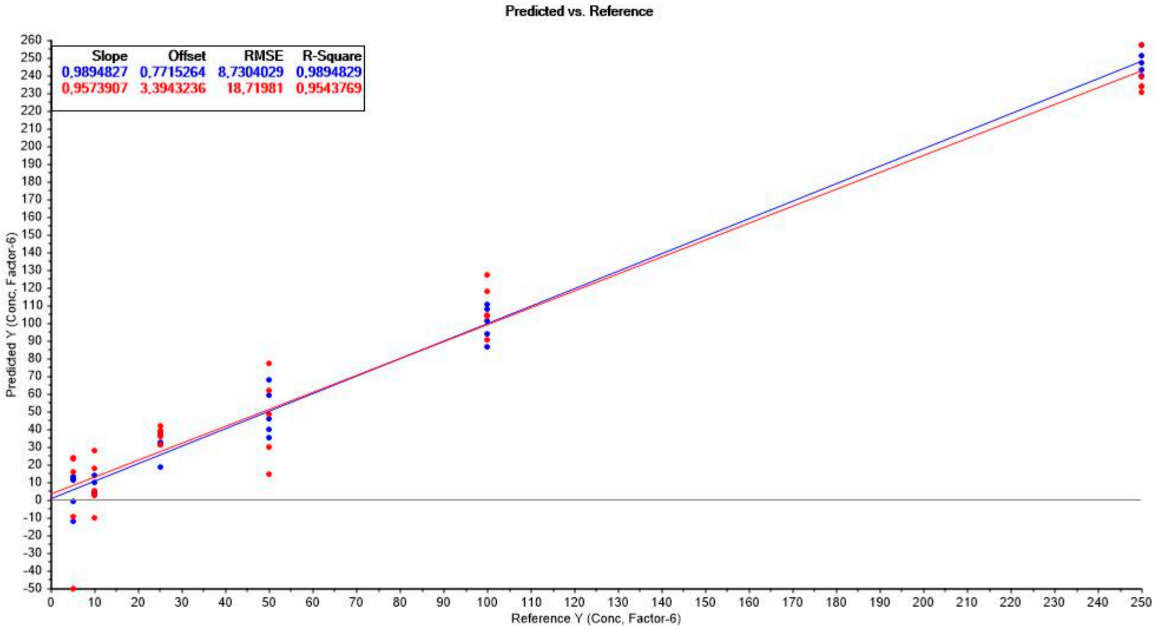

Figure S10c. Observed and predicted values of iprodione concentrations in red wine using 6 factors. Coefficient of determination R^2_C for calibration (blue) and R^2_V for validation (red) datasets, root mean squared errors (RMSEC for calibration set and RMSEV for validation set). Iprodione concentrations varied between 0 and 250 ng.L^{-1}.

Estimation of LOD

Results for red wine:

var(x) [(ng/L)^2]	var(ycal))	h_{0min}	h_{0max}	SEN
0.0003	73.917	0.110	0.489	0.036
